# Barriers and facilitators in utilisation of dental health services across low- and middle-income countries: a scoping review

**DOI:** 10.1038/s41432-025-01200-0

**Published:** 2026-01-13

**Authors:** Priyanshu Kumar Shrivastava, Abhishek Mehta, Barsha Priya Deka, Manu Raj Mathur

**Affiliations:** 1 Postgraduate student, Department of Epidemiology and Public Health, University College London, London, WC1E 6BT UK; 2 Department of Public Health Dentistry, Faculty of Dentistry, Jamia Millia Islamia, New Delhi, 110025 India; 3 Department of Dental Public Health and Primary Care, Institute of Dentistry, Queen Mary University of London, London, E1 2AD UK

**Keywords:** Health care, Dentistry

## Abstract

**Objectives:**

This scoping review aims to systematically identify and theoretically categorise barriers and facilitators affecting access to dental care in LMICs.

**Methods:**

This scoping review employed the Theoretical Domains Framework (TDF) to synthesise the findings thematically. A comprehensive search of literature published up to May 2025 in MEDLINE (PubMed), Embase, Scopus, and Web of Science, as well as grey literature sources, was conducted to identify relevant articles. Screening was performed using Rayyan, and data were extracted and categorised into the TDF domains.

**Results:**

From the 15,140 initial hits, 214 articles were found eligible for final analysis. The maximum number of studies was published in India, Brazil, Nigeria and Iran. Eleven TDF domains were identified, and ‘environmental context and resources’ (*n* = 452, 41.5%) emerged as the most frequent domain, followed by ‘beliefs about consequences’ (*n* = 251, 23.1%) and ‘knowledge’ (*n* = 144, 13.2%). Barriers were more frequently reported than facilitators across all domains. Distinct domain patterns were observed across population subgroups and income categories, with structural barriers dominating in all contexts.

**Conclusion:**

Findings from this review underscore the need for integrated, context-sensitive interventions that combine system-level reforms with behaviour change strategies to improve dental care utilisation and reduce inequalities in oral health access across LMICs. There is also a need for more research on this health issue in low-income countries.

Key Points

- Access to dental care in LMICs is primarily shaped by environmental and structural constraints, underscoring the need for financing reforms and stronger integration of oral health into primary care.
- Utilisation patterns in LMICs remain largely reactive rather than preventive, contributing to higher out-of-pocket expenses and adding to the burden on already strained health systems.
- One-size-fits-all response is not feasible. Upper-middle-income countries should focus on consolidating oral health within universal health coverage frameworks, while lower-income settings must prioritise basic infrastructure, affordability, and targeted interventions for marginalised populations.

## Introduction

Preventable yet widespread, oral diseases represent one of the most overlooked global health challenges^1^. Their impact is especially significant among individuals living in low- and middle-income countries (LMICs), where access to dental care is marked by profound inequities arising from systemic challenges^2^. An underdeveloped public health infrastructure, a shortage and uneven distribution of dental professionals, urban-centric service delivery models, poor integration of oral health within the broader health system, limited or no insurance coverage, and minimal public funding for oral health services are a few of the existing challenges^3^.

To strengthen the oral health care access, availability and affordability in LMICs, it is essential to integrate it with the existing primary health care systems^4^. However, this integration cannot occur without first identifying and addressing access-related barriers and promoting existing facilitators. Understanding the root causes of these barriers and enablers is crucial for reducing inequities, optimising resource allocation, designing effective policies and interventions, and achieving universal oral health coverage^5^.

Much of the existing literature primarily describes the utilisation patterns without exploring the underlying mechanisms that drive individual care-seeking decisions. These barriers or facilitators stem from a complex interplay of personal, cultural, social and environmental factors^5,6^. Analysing these determinants through a theory-driven framework is essential for identifying modifiable factors and enhancing the design of effective interventions.

This Theoretical domain framework (TDF) was used to guide this review because it provides a comprehensive, evidence-based structure for identifying the behavioural determinants of healthcare utilisation. The TDF integrates key constructs from 33 behavioural change theories into 14 domains, encompassing both internal (physical or psychological) and external (environmental) influences on behaviour^7^. It has been widely applied across public health settings to identify barriers and facilitators to health-seeking behaviour and inform behavioural interventions^8,9^. In the LMIC context, where utilisation is shaped by complex interactions between systemic constraints and individual beliefs, the TDF provided a systematic and theory-driven approach to map the influences and highlight modifiable targets for intervention.

A substantial body of research has examined the determinants influencing utilisation of dental health care globally, generating a volume of data. Although several reviews have attempted to synthesise this evidence, their scope is limited as they focus on specific population subgroups^10–12^. Further, these reviews aggregate data from countries across all income levels, without isolating the unique contextual factors related to LMICs^2^. Hence, a critical gap exists in the literature due to the absence of a comprehensive evidence synthesis on this issue, particularly through the lens of a theory-driven behavioural framework. To address this lacuna, we conducted a scoping review to map the existing evidence on barriers and facilitators influencing the utilisation of oral health services in LMICs using a theoretical framework.

## Material and methods

A scoping review methodology was chosen, given the broadness of the review question and diversity of study designs, populations, and contexts. The review followed Arksey and O’Malley’s five-stage framework and the PRISMA-ScR (Preferred Reporting Items for Systematic Reviews and Meta-Analyses Extension for Scoping Reviews) checklist^13,14^. A detailed protocol was registered on the Open Science Framework^15^. The research question was: “What are the behavioural, contextual barriers and facilitators that influence the use of dental care services across different population groups in LMICs?”

### Eligibility criteria

We utilised the PERSPECT (Perspective, Evaluation Focus, Research Type, Setting, Population, Exclusions, Comparators, and Timeframe) framework to shortlist the studies for this review. The details for the eligibility criteria are as follows:

Perspective (P): Studies reporting the perspectives of patients, caregivers, or service users of dental care were included.

Evaluation Focus (E): Studies assessing barriers and/or facilitators to accessing, seeking, or utilising dental care were included.

Research Type (R): Qualitative and quantitative approaches (cross-sectional, cohort, or case-control), or mixed-methods designs were included. Editorials, commentaries, opinion pieces, and conference proceedings were excluded.

Setting (S): Community or clinical settings in LMICs defined by the World Bank classification were eligible^16^. High-income countries or multi-country studies without LMIC-specific data were excluded.

Population (P): Included were general adults and special groups (e.g. older adults, persons with disabilities, children, pregnant women). Studies limited to healthcare providers or administrators were excluded.

Exclusions (E): Studies not assessing barriers or facilitators, or focused on clinical outcomes for specific treatments, and studies lacking a sufficient methodological approach were excluded.

Comparators (C): No restrictions were placed on comparators.

Timeframe (T): Studies published up to May 2025 were considered.

### Search strategy

A comprehensive search was conducted to identify published and unpublished literature up to May 2025. An initial search of PubMed and Scopus was performed to identify relevant reviews or studies and develop a final search strategy using the shortlisted keywords (Supplementary Table S1). The electronic databases used were MEDLINE (PubMed), Embase, Scopus, and Web of Science. Google Scholar, ProQuest, and Open Grey were searched to check for any grey literature available. Reference lists of relevant articles were screened. No language restrictions were imposed, and non-English articles were translated to English using DeepL (DeepL SE, Cologne, Germany)^17^.

### Study selection

All citations were uploaded into Rayyan (Rayyan Systems Inc.), and duplicates were removed^18^. Two reviewers (PKS, BPD) independently screened the titles and abstracts of the articles, and the same reviewers conducted the screening of the full text of eligible studies. Any disagreements at any stage were resolved through discussion or consultation with a third reviewer (AM).

### Data charting

Data from eligible studies were extracted independently by two reviewers using a pre-tested Excel spreadsheet. Pre-testing was done on ten randomly selected studies, with discrepancies resolved by a subject expert (AM). Extracted information included study ID, author, title, year, study design, methodological approach, and country classified as per World Bank income into Low-income (LI), Lower-middle-income (LMI), or Upper-middle-income (UMI)^16^, population characteristics, sample size, and study setting. Reported dental service utilisation rates were documented where available. For each study’s three key barriers and facilitators were identified and mapped to corresponding TDF domains.

### Data analysis and presentation

The barriers and facilitators were categorised into specific domains of TDF, a comprehensive model that integrates constructs from multiple behavioural theories into fourteen domains^7,19^. The domains include intentions, decision-making processes, goals, emotions, environmental context and resources, reinforcement, social influences, memory, attention, behavioural regulation, social/professional role, optimism, beliefs about capabilities, and beliefs about consequences^7^. Detailed analyses of each domain were conducted to highlight the key barriers and facilitators that influence access to dental services. Furthermore, a subgroup analysis was conducted to identify country-specific barriers and facilitators among different population groups.

## Results

### Study selection

**Fig. 1:**
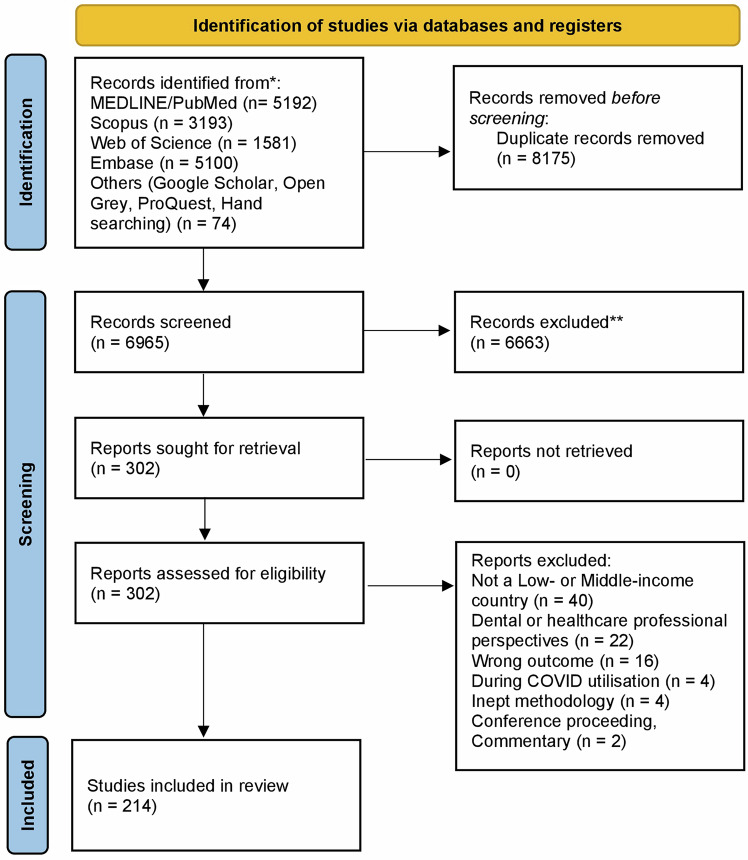
PRISMA 2020 flowchart for study selection process.

Our initial search yielded a total of 15,140 citations (MEDLINE/PubMed = 5192; Scopus = 3193; Web of Science = 1581; Embase = 5100; Grey literature = 74). After removing duplicates (*n* = 8175), the title and abstract of the remaining (*n* = 6965) articles were screened using Rayyan software. Full-text review of 302 articles was performed, and 88 articles were excluded for reasons such as high-income country populations, provider-only perspectives, or lack of access determinants. Finally, 214 articles met the inclusion criteria and were incorporated into this scoping review (Supplementary Table S2). Figure 1 provides a summary of the selection process following PRISMA guidelines.

### Study characteristics

**Fig. 2:**
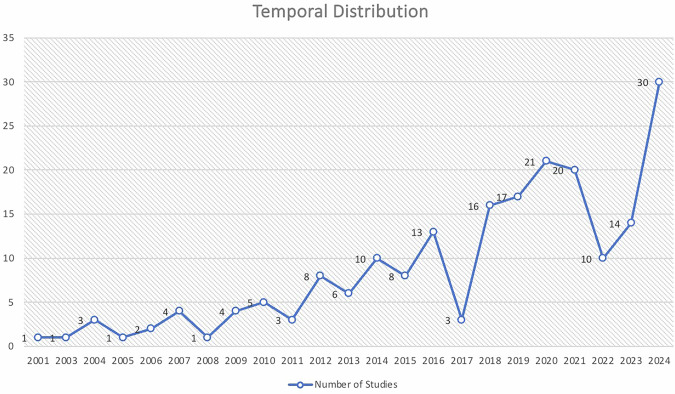
Temporal distribution of studies.

Data from 214 studies conducted in 34 LMICs was included in this review. One of the study was a multi-country study which reported data from 1 UMI and 4 LMI countries, resulting in 218 country-level entries^4^. Based on these entries, 106 were from UMI countries, 104 from LMI countries, and only eight from LI countries. India accounted for the highest number of studies, followed by Brazil, Iran, and Nigeria (Table 1). Progressive increase in the number of studies from 2001 onwards was observed, with the peak annual output recorded in 2024 (*n* = 30) (Fig. 2). The cumulative sample comprised 701,090 participants, comprising 221,071 males and 229,236 females, with the remainder not mentioning gender-wise distribution. The age groups spread from infants to 103 years. Most studies focused on general adult populations, followed by children and older adults. Special population groups included pregnant women, children with special healthcare needs, individuals with disabilities, HIV-positive and transgender populations, with older adult and disability studies concentrated in UMI countries (Table 1).

**Table 1:** Distribution of included studies by income category and population groups

Category	Upper-middle income (*n* = 106)	Lower-middle income (*n* = 104)	Low income (*n* = 8)	Total
Number of countries	15	14	5	34
Countries with number of entries	Brazil (36), Iran (18), Thailand (9), China (8), Malaysia (6), Mexico (6), Jordan (5), Colombia (3), Indonesia (3), Turkey (3), Ecuador (2), Lebanon (2), Peru (2), South Africa (2), Fiji (1)	India (61), Nigeria (18), Pakistan (8), Nepal (4), Tanzania (3), Vietnam (2), Angola (1), Bangladesh (1), Cambodia (1), Egypt (1), El Salvador (1), Kenya (1), Lesotho (1), Zimbabwe (1)	Uganda (4), Burkina Faso (1), Congo Dem. Rep. (1), Ethiopia (1), Sudan (1)	34 countries with 218 entries
Population groups				
General adult population	31	52	4	87
Children and adolescents	18	14	None reported	32
Older adults	15	7	None reported	22
Pregnant women	13	6	1	20
Children with special health care needs	8	9	2	19
Persons with disabilities	10	1	None reported	11
University staff and students	3	6	None reported	9
Individuals living with HIV	1	3	1	5
Transgender adults	1	4	None reported	5
Children with dental problems	2	1	None reported	3
Sex workers	1	None reported	None reported	1
Individuals with inherited bleeding disorders	1	None reported	None reported	1
Individuals with leukaemia	1	None reported	None reported	1

Majority of study followed cross-sectional design (*n* = 177), the rest were case-control (*n* = 5), qualitative (*n* = 24), mixed-method (*n* = 7), and only one was longitudinal^20^. Most studies were conducted in a community-based setting (*n* = 99), including 15 nationwide surveys, followed by public health or dental care centres (*n* = 69), schools or universities (*n* = 34), and a smaller number from private facilities (*n* = 3) and special care homes (*n* = 9). Data analyses commonly involved descriptive statistics (*n* = 111), regression (*n* = 75), and thematic or content analysis (*n* = 28). Few studies explicitly applied theoretical models to assess the barriers and facilitators, most commonly Anderson’s behavioural model (*n* = 53), followed by Penchansky & Thomas’ Five-As Access Model (*n* = 4), Jean-Frederic Levesque’s access model, Predisposing, Reinforcing, and Enabling Constructs in Educational Diagnosis and Evaluation (PRECEDE) framework, and Bradshaw’s model of health needs^21–23^ (Supplementary Table S2).

### Pattern of dental services utilisation

Overall average prevalence of dental services utilisation, encompassing both regular and occasional dental care visits, was 30.35% while 47.09% participants had never utilised dental care in their lifetime. Utilisation rates varied slightly across income groups, with the highest rates reported in lower-middle-income countries (31.15%), followed by upper-middle-income (27.12%) and low-income countries (24.08%). The most common reason for seeking dental care, reported in 60 studies, was emergency-care situations, such as pain or swelling. This was followed by curative services (*n* = 57), including dental treatment procedures like extractions, restorations, periodontal or orthodontic interventions, and aesthetic care. Routine dental check-ups (*n* = 7) and preventive services (*n* = 5) were infrequently cited as reasons for utilisation. Public health centres emerged as the preferred point of care in most studies (*n* = 57), although a notable number reported a preference for private facilities (*n* = 27) (Supplementary Table S2).

### TDF domain analysis

**Fig. 3:**
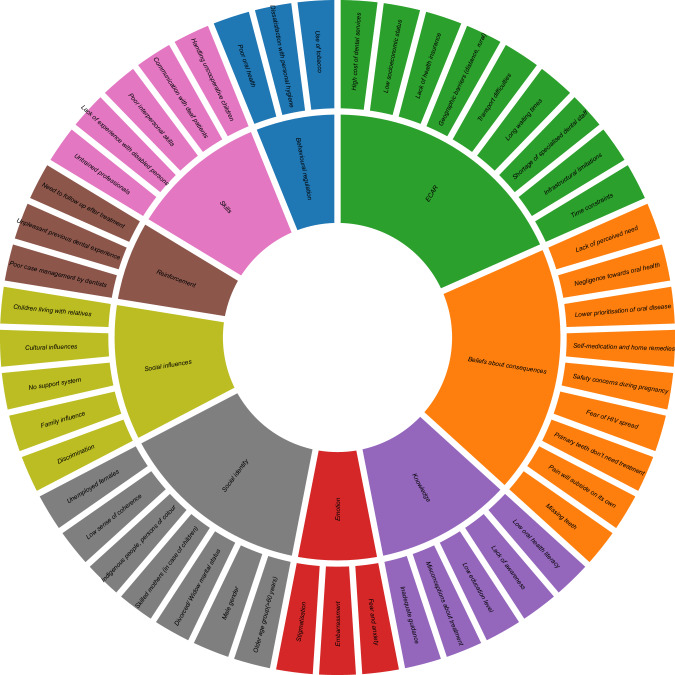
Barriers to dental service access in LMICs aligned with the TDF domains. *ECAR Environmental context and resources.

**Fig. 4:**
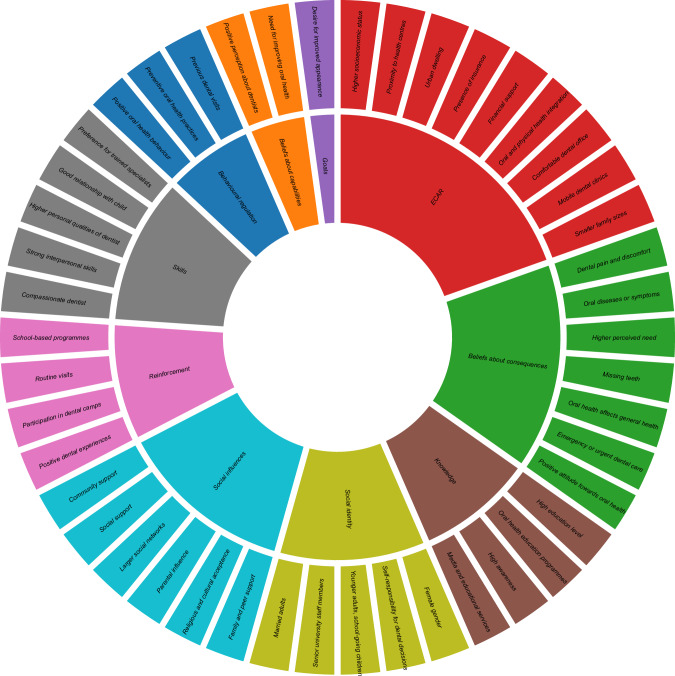
Facilitators to dental service access in LMICs aligned with the TDF domains. *ECAR Environmental context and resources.

Across 11 TDF domains, *“environmental context and resources”* (*n* = 452, 41.5%; barriers: *n* = 301, 48.9%; facilitators: *n* = 151, 31.9%) was most prominent, followed by *“beliefs about consequences”* (*n* = 251, 23.1%; barriers: *n* = 118, 19.1%; facilitators: *n* = 133, 28.1%) and “*knowledge”* (*n* = 144,13.2%; barriers: *n* = 75, 52%; facilitators: *n* = 69, 47.9%). *“emotion”* (*n* = 55, 5%) comprised barriers only, while facilitators were more common in *“social influences”, “reinforcement”, “behavioural regulation”, and “belief about capabilities”*. Figures 3 and 4 illustrate the key barriers and facilitators observed across the domains, with the overall distribution and further stratification by income level and population group in Supplementary Figs. S1–S3.

In UMI countries, barriers were dominated by *“environmental context and resources”*. Older adults faced physical limitations, low perceived need, and cultural norms, while pregnant women reported safety fears of dental procedures during pregnancy, and the absence of childcare support systems or creches. Persons with disabilities encountered inaccessible infrastructure and inadequate provider training, and those with inherited bleeding disorders expressed distrust in non-specialised providers. Facilitators were more prominent within the “*environmental context and resources”* domain, including urban residence and higher socioeconomic status (SES). School-based programs and access to paediatric specialists were facilitators for children, while for older adults, insurance schemes, proximity to health centres, and prosthetic needs such as dentures were enablers. For pregnant women, previous pregnancy experience and symptom-driven care-seeking encouraged service uptake. Children with special healthcare needs and persons with disabilities preferred integrated hospital care and communication-friendly professionals. Other groups, such as sex workers, often sought dental services for aesthetic reasons or infection control. Facilitators under the “*knowledge”* domain, such as education campaigns and media exposure, were more frequently observed in UMI countries, and the “*skills”* domain, referring to provider competencies, was also more prominent in these countries.

In LMI and LI countries, barriers were more widespread, dominated by structural issues such as high costs, low SES, lack of insurance, geographic inaccessibility, transport difficulties, and low perceived need. Social identity barriers such as male gender, minority status were also frequently documented. For children and adolescents, low household income, lack of transport, poor referral follow-up, and parental beliefs, combined with low education levels, restricted utilisation. Most studies on transgender individuals and people living with HIV emerged from LMI countries, and stigma and discrimination created significant barriers in this group, while inclusive facilities for the transgender community and symptom-based care seeking for HIV- positive people were important facilitators. Among rural populations, proximity to clinics and pain-driven care-seeking were key facilitators. The *“Beliefs about consequences”* domain was especially prominent in LMI countries, reflecting diverse oral health beliefs and behaviour. Poor awareness under the *“knowledge domain”* was common in LMI/LI countries, although some community-based education initiatives served as facilitators. Barriers related to *“emotional”* and “*social influences”* were also more evident, with cultural norms and family or peer influence shaping behaviour. Facilitators associated with the *“reinforcement”* and *“beliefs about capabilities”* domains emerged through community-based programs and increased confidence in accessing care. In LI countries, patterns closely resembled those of LMI countries, with no notable differences observed.

## Discussion

This scoping review systematically mapped barriers and facilitators affecting dental service use in LMICs using the TDF framework. The identified barriers and facilitators were closely connected, reflecting systemic issues like weak health governance, uneven distribution of the workforce, and socio-economic inequalities. With 214 studies published on this topic, it’s clear that research on dental service utilisation in LMICs has grown, especially over the last twenty years. However, only eight studies focused on low-income countries, highlighting their significant under-representation in the evidence base. This demonstrates broader disparities in global health research, where countries most in need often have the least visibility. The patterns emerging from this review highlight a striking consistency across various LMIC settings. Our findings confirm that oral health remains a largely overlooked public health issue in many LMICs, despite its significant impact on populations. In line with global reports, national health strategies continue to allocate minimal funding and policy focus to oral health. Dental service delivery systems remain fragmented, underfunded, and heavily concentrated in urban centres, where private providers primarily deliver care. This urban bias worsens disparities, leaving rural and marginalised populations with limited or no access to affordable and timely care.

The dominance of the *“environmental context and resources”* domain underscores that structural determinants remain the primary levers for improving equitable access in LMICs. High treatment costs, limited insurance, workforce maldistribution, long travel distances, and inadequate service organisation consistently emerged as key barriers, aligning with global evidence that resource inequities and high Out-Of-Pocket Expenses (OOPE) are key drivers of oral health inequities^24,25^. The issues are compounded by the fact that ~88% of global oral health expenditure occurs in high-income countries, serving just over one-fifth of the world’s population^26^. Low perceived need emerged recurrently as a barrier, reflecting low prioritisation of oral health, while dental pain as a primary motivator indicated that access is largely reactive in LMI settings. This utilisation pattern further contributes to higher OOPE, poorer health outcomes, and an escalating burden on already strained health systems^27^.

Knowledge-related barriers, especially low oral health literacy, reinforce the need for integration of oral health into schools, community outreach, and media campaigns. Emotional barriers such as fear and anxiety, compounded by stigma and discrimination toward marginalised groups, underscore the need for stigma reduction and culturally competent, inclusive care. Less frequently reported domains, such as provider skills, reinforcement, and behavioural regulation, indicate that, when present, factors like quality-of-care experiences and entrenched habits can shape utilisation patterns. Addressing misconceptions about oral health, particularly among parents and pregnant women, as noted in previous reviews, is essential^10,28^. These patterns are broadly consistent with previous oral health research in high-income countries, where environmental and knowledge domains also dominate but differ in LMICs by the greater salience of health-related behaviour and social influences^29^.

Across diverse LMICs, country experiences show similar patterns; policy reforms have delivered partial gains in dental access but have not fully addressed deep-rooted inequities. Brazil’s 2004 National Oral Health Policy (Política Nacional de SaúdeBucal, PNSB) decentralised services and prioritised pain and infection management; however, persistent challenges with funding and sustainability continue to limit its full impact^30^. In India, despite policy provisions, primary health centres, the sole public health facilities in many rural areas, lacked dental practitioners^31^. In Nigeria, high OOPE and lack of public insurance exacerbated rural inequities, with third-party payment systems influencing utilisation^32^. Comparable financial barriers were also reported in other African and Asian contexts.

While studies from UMI countries uncovered more system-level enablers, the LMI and LI countries remained reliant on symptom-driven care and prevailing health beliefs, implying that a one-size-fits-all response is not feasible. Higher-income LMICs should consolidate financing reforms and integrate oral health into universal health coverage (UHC) while strengthening primary-care, whereas LMI settings need combined supply and demand-side approaches like workforce deployment, health literacy, stigma reduction, community outreach, and LI countries must prioritise basic infrastructure, affordability, and strategically distributed services.

Sustained investment in affordable, publicly funded oral health services is central to LMIC settings, with integration into primary healthcare systems as advocated by the WHO Global Oral Health Action Plan (2023–2030)^33^. Experiences from Thailand and Brazil demonstrate how incorporating dental care into UHC can enhance accessibility^34,35^, while strategies such as task-shifting to mid-level providers, deployment of community health workers, and mobile dental clinics can bridge geographic and workforce gaps^36,37^. Policymakers should adopt multifaceted “barrier-guided” interventions, for instance, by expanding insurance or fee-exemption schemes to improve affordability and accessibility, and educational campaigns and community engagement to mitigate knowledge gaps and shape health beliefs. Applying an equity lens and implementing targeted interventions for marginalised groups guided by a robust framework, such as PROGRESS (Place, Race, Occupation, Gender, Religion, Education, Socioeconomic Position), is mandated to avoid worsening inequities^38^.

Further research is particularly needed in LI countries and marginalised populations to address existing evidence gaps. Longitudinal and implementation research is required to test whether integrating dental care into PHC, expanding insurance, or running education programs boost utilisation and improve outcomes. Economic evaluations or cost-effectiveness studies would help policymakers prioritise resource allocation. Applying behavioural frameworks like the TDF can strengthen the evidence base by linking specific interventions to the underlying behavioural determinants.

A major strength of this review is its comprehensive scope, synthesising from 214 studies conducted across diverse LMICs, and the application of a robust behavioural framework makes this one of the first large-scale uses of the TDF to oral health access. Limitations include potential ambiguity in coding certain barriers, like age or gender, that can be both contextual and identity-related, and the risk of losing contextual nuance due to single deductive coding, though specific reasons were quoted in each domain to mitigate this. Studies varied widely in methods, sampling and outcome measures, increasing heterogeneity and limiting comparability. A smaller number of studies from LI countries further restricts generalisability to these settings.

## Conclusion

In LMICs, access to dental health services is shaped predominantly by structural and economic constraints; however, beliefs, knowledge, and social factors play equally important roles. Achieving equitable access, therefore, requires multi-level strategies that combine systemic reforms such as improving infrastructure, expanding financial protection, and integrating oral health into primary care with community-level interventions that address health literacy, cultural perceptions, and behavioural barriers.

The expanding evidence base and recent global policy frameworks, including the WHO Global Oral Health Action Plan, provide a clear direction for action. However, persistent research and implementation gaps, especially in LI countries with the highest unmet need, must be addressed with urgency. Ensuring universal access to oral healthcare is not only a public health imperative but also a key component of achieving global health equity.

## Supplementary information

Supplementary figures S1,S2,S3

Detailed search strategy

Detailed study characteristics

PRISMA ScR Checklist

## Data Availability

The data supporting the findings of this study are available in the supplementary material of this article.
