# Supplementary material for: Barriers and facilitators in utilisation of dental health services across low- and middle-income countries: a scoping review
Source: Evid Based Dent. 2026 Jan 13;27(1):19. doi: 10.1038/s41432-025-01200-0 (PMC13031122; doi:10.1038/s41432-025-01200-0)
Supplement: Supplementary file 1 — Supplementary figures S1,S2,S3 [file 41432_2025_1200_MOESM1_ESM.docx]

**Supplementary Figures**

**
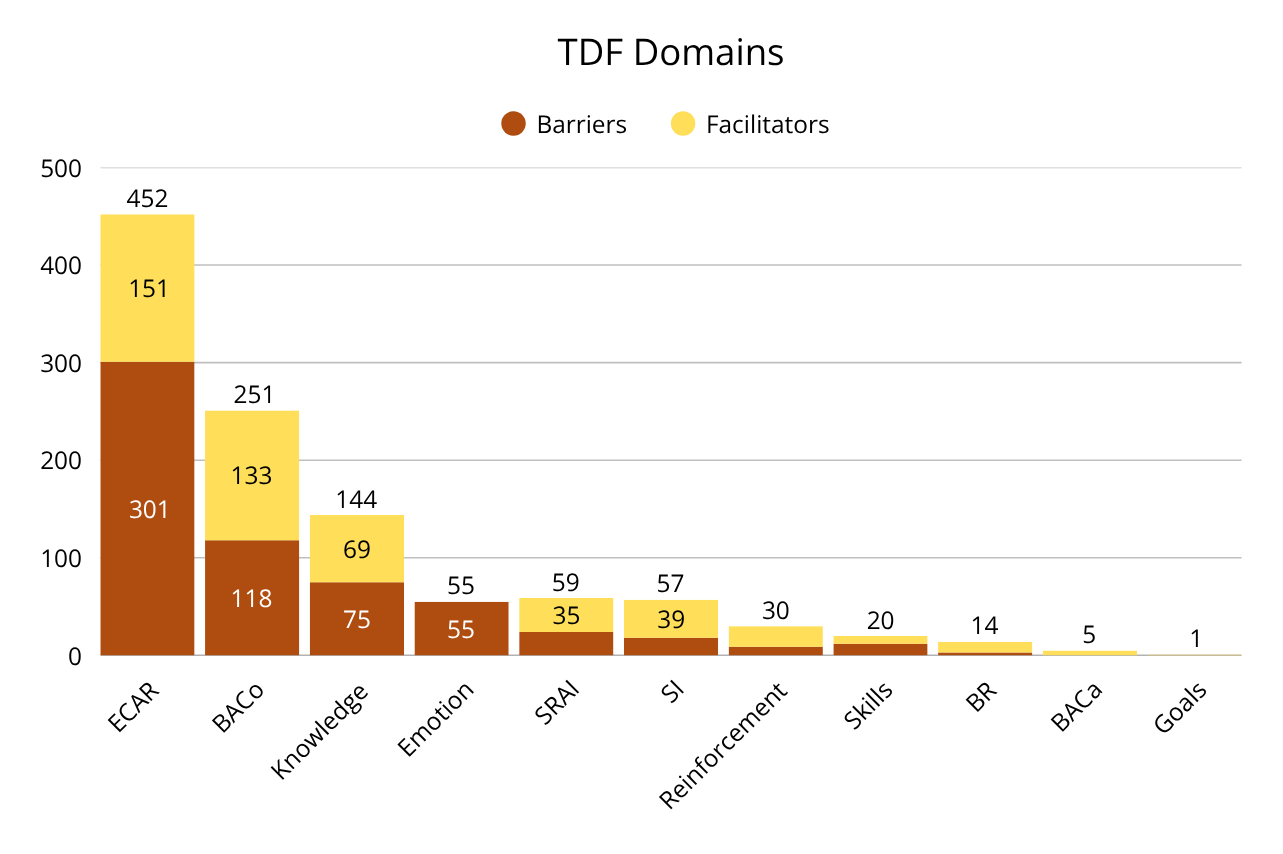
**

Figure S1: Overall distribution of barriers and facilitators across the TDF domains. ECAR: Environmental context and resources, BACo: Beliefs about consequences, SRAI: Social/professional role and identity, SI: Social influences, BR: Behavioural regulation, BACa: Beliefs about capabilities


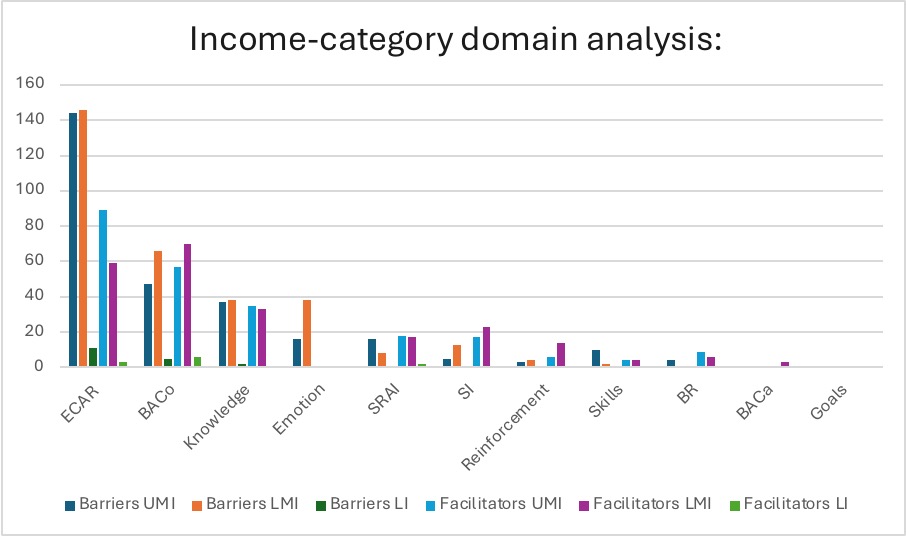


Figure S2: Barriers and facilitators across the TDF Domains with income-level comparison. ECAR: Environmental context and resources, BACo: Beliefs about consequences, SRAI: Social/professional role and identity, SI: Social influences, BR: Behavioural regulation, BACa: Beliefs about capabilities, UMI: Upper-middle income, LMI: Lower-middle income, LI: Low-income


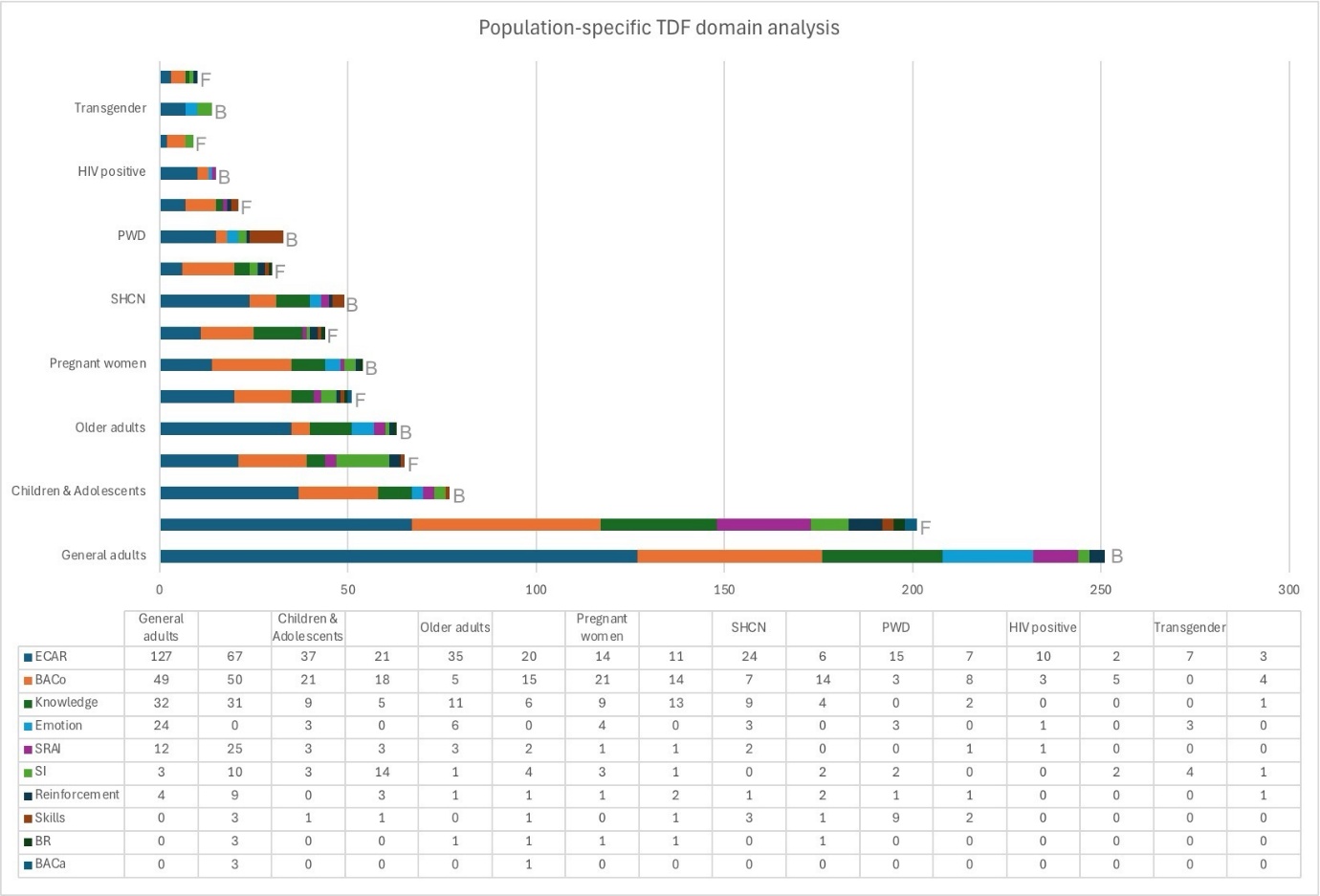


Figure S3: Distribution of TDF domains across the population subgroups. ECAR: Environmental context and resources, BACo: Beliefs about consequences, SRAI: Social/professional role and identity, SI: Social influences, BR: Behavioural regulation, BACa: Beliefs about capabilities, SHCN: Special Healthcare needs, PWD: Persons with disabilities.
