# Supplementary material for: Barriers and facilitators in utilisation of dental health services across low- and middle-income countries: a scoping review
Source: Evid Based Dent. 2026 Jan 13;27(1):19. doi: 10.1038/s41432-025-01200-0 (PMC13031122; doi:10.1038/s41432-025-01200-0)
Supplement: Supplementary file 2 — Detailed search strategy [file 41432_2025_1200_MOESM2_ESM.pdf]

Search Strategy

## MEDLINE/Pubmed:

| Search statement | Search terms | Results |
|---|---|---|
| #1 | ("barrier"[All Fields] OR "barrier s"[All Fields] OR "barriers"[All Fields]) OR ("challenge"[All Fields] OR "challenged"[All Fields] OR "challenges"[All Fields] OR "challenging"[All Fields]) OR ("obstacle"[All Fields] OR "obstacles"[All Fields]) OR ("hindrance"[All Fields] OR "hindrances"[All Fields]) OR ("facilitate"[All Fields] OR "facilitated"[All Fields] OR "facilitates"[All Fields] OR "facilitating"[All Fields] OR "facilitation"[All Fields] OR "facilitations"[All Fields] OR "facilitative"[All Fields] OR "facilitator"[All Fields] OR "facilitator s"[All Fields] OR "facilitators"[All Fields]) OR ("motivate"[All Fields] OR "motivated"[All Fields] OR "motivates"[All Fields] OR "motivating"[All Fields] OR "motivation"[MeSH Terms] OR "motivation"[All Fields] OR "motivations"[All Fields] OR "motive"[All Fields] OR "motivational"[All Fields] OR "motivator"[All Fields] OR "motivators"[All Fields] OR "motives"[All Fields]) OR ("enable"[All Fields] OR "enabled"[All Fields] OR "enablement"[All Fields] OR "enablements"[All Fields] OR "enabler"[All Fields] OR "enablers"[All Fields] OR "enables"[All Fields] OR "enabling"[All Fields])) | 3490412 |
| #2 | ("dental care"[All Fields] OR "dental healthcare"[All Fields] OR "dental care delivery"[All Fields] OR "dental health services"[All Fields] OR "dental visit"[All Fields] OR "dental checkup"[All Fields] OR "dental treatment"[All Fields]) | 58515 |
| #3 | ("access"[All Fields] OR "accessed"[All Fields] OR "accesses"[All Fields] OR "accessibilities"[All Fields] OR "accessibility"[All Fields] OR "accessible"[All Fields] OR "accessing"[All Fields] OR ("access"[All Fields] OR "accessed"[All Fields] OR "accesses"[All Fields] OR "accessibilities"[All Fields] OR "accessibility"[All Fields] OR "accessible"[All Fields] OR "accessing"[All Fields]) OR ("statistics and numerical data"[MeSH Subheading] OR ("statistics"[All Fields] AND "numerical"[All Fields] AND "data"[All Fields]) OR "statistics and numerical data"[All Fields] OR "utilization"[All Fields] OR "utilisation"[All Fields] OR "utilisations"[All Fields] OR "utilise"[All Fields] OR "utilised"[All Fields] OR "utilises"[All Fields] OR "utilising"[All Fields] OR "utilities"[All Fields] OR "utility"[All Fields] OR "utilizations"[All Fields] OR "utilize"[All Fields] OR "utilized"[All Fields] OR "utilizer"[All Fields] OR "utilizers"[All Fields] OR "utilizes"[All Fields] OR "utilizing"[All Fields])) | 5376556 |
| #4 | #1 AND #2 AND #3 | 5192 |
