# Supplementary material for: Barriers and facilitators in utilisation of dental health services across low- and middle-income countries: a scoping review
Source: Evid Based Dent. 2026 Jan 13;27(1):19. doi: 10.1038/s41432-025-01200-0 (PMC13031122; doi:10.1038/s41432-025-01200-0)
Supplement: Supplementary file 3 — Detailed study characteristics [file 41432_2025_1200_MOESM3_ESM.pdf]

S. No	First Author	Title	Year	Country of Study	World Bank Classification	Study design	Sampling approach	Methodological approach	Setting	Theoretical framework used	Population type	Informant (Patient/ Caregiver)	Sample size	Dental Service utilisation (n)	Dental Service utilisation (%)	Primary reason for utilisation	Place of last visits	Never visited (n)	Never visited (%)	Age (Range /mean/ median in years)	Male	Female
1	Abdollahi M	Perceptions and determinants of oral health care among Iranian pregnant women: a qualitative study.	2024	Iran	UMI	Qualitative	Purposive sampling	Content analysis with a guided approach using SE and Shannon's method	Health service centres	Thematic analysis	Pregnant women	Self-reported	18	.	.	.	.	.	.	20-44	.	18
2	Abodunrin OR	Factors associated with the dental service utilization by enrollees on the Lagos State health insurance scheme, Nigeria	2025	Nigeria	LMI	Cross Sectional	Stratified random sampling	Univariate and Multivariate analysis with Binary logistic regression	Community Based Survey (Lagos)	Delphi method used for tool validation	General adults	Patient	485	31	0.064	Emergency and Need Based Dental Care	Public or Government based dental service	454	0.936	18-72	237	248
3	Adedigba MA	Pattern of Utilisation of Dental Health Care Among HIV-positive Adult Nigerians.	2016	Nigeria	LMI	Cross-sectional	Convenience sampling	Bivariate and Multivariate logistic regression analysis	Health service centres	NR	HIV-positive adults	Self-reported	239	.	.	.	.	223	93.30%	31-40	85	154
4	Adeniyi AA	Predisposing, enabling and need factors influencing dental service utilization among a sample of adult Nigerians	2020	Nigeria	LMI	Cross-sectional	Multi-stage cluster random sampling	Bivariate and Multivariate logistic regression analysis	Urban and Rural community-based survey	Anderson and Newman	General adults	Self-reported	400	43	0.108	.	.	243	0.608	18-80 (35.51 ± 11.16)	168	232
5	Adriani M	Determinants of the Utilization of Dental and Oral Health Care Services by Pregnant Women in Pancoran Mas Health Center Depok City	2022	Indonesia	UMI	Cross-sectional	Multi-stage random sampling	Bivariate and Multivariate logistic regression analysis	Household survey	NR	Pregnant women	Self-reported	162	42	0.259	.	.	120	0.741	13-30	.	162
6	Ahmad W	Barriers in Access and Utilization of Dental Care: Assessment and Recommendations Using Delphi Technique	2019	Pakistan	LMI	Cross Sectional and Delphi Technique	Multi Stage random Sampling	Mixed Method	Urban community based survey	Delphi method used for recom	General adults	Patients	535	NR	NR	NR	NR	43	0.08	Mean 30. 6	355	180

										mendation												
7	Aikins EA	Utilization of dental services among civil servants in Port Harcourt, Nigeria	2015	Nigeria	LMI	Cross Sectional	Multi Stage random Sampling	Descriptive and Chi-Square Test	Urban community based survey	NR	General adults (civil servants)	Patients	638	143	0.224	Curative Dental Care	Government dental services	49	0.776	17-65	326	312
8	Ajayi DM	Barriers to oral health care utilization in Ibadan, South West Nigeria.	2012	Nigeria	LMI	Cross-sectional	Convenience sampling	Descriptive statistics for ranking barriers	Hospital-based survey	NR	General adults	Self-reported	400	.	.	Pain (55.8%)	.	109	0.273	16-78 (37.85 ± 15.38)	78	222
9	Ajayi EO	Utilization of dental services in a population of Nigerian University Students	2007	Nigeria	LMI	Cross Sectional	Simple random sampling	Quantitative with structured questionnaire	University based suvery	NR	University students	Patients	375	53	0.141	Curative Dental Care	NR	322	0.859	17-33	245	130
10	Akram SJ	Barriers to the access of oral health care facilities among adults: an exploratory study from Lahore	2020	Pakistan	LMI	Cross-sectional	Purposive sampling	Descriptive statistics for ranking barriers and Chi-square for association	Hospital-based survey	NR	General adults	Self-reported	400	187	0.4675	Checkup (75%), Emergency (19.75%)	Private services (58.50%), Subsidized facilities (30.25%)	21	0.0525	18-63 (36.81 ±9.29)	200	200
11	Al Habashneh R	Oral health status and reasons for not attending dental care among 12- to 16-year-old children with Down syndrome in special needs centres in Jordan.	2012	Jordan	UMI	Case-control	NR	Percentages	Hospital-based survey	NR	Children with Down syndrome	Mother	206	45	.	Extraction	.	81		13.66 ± 1.47	136	70
12	Alade GO	Self-reported Treatment Needs and Utilization of Dental Services among Dental Students and Dental Technology Students	2022	Nigeria	LMI	Cross-sectional	NR	Percentages	Hospital-based survey	NR	Dental students and Dental technology students	Self-reported	219	112	51.10%	Treatment (Scaling and Polishing)	.	107	48.90%	16-42 (23.3 ± 3.8)	74	145
13	Aliuddin AM	Obstacles to utilization of dental services in karachi, pakistan	2021	Pakistan	LMI	Cross-sectional	Non probability consecutive sampling	Percentages	Hospital-based survey	NR	General adults	Adults	500	.	.	.	.	94	.	20-60	188	312

14	Alshatrat S	Oral health knowledge, behaviour, and access to dental care in visually impaired individuals in jordan: A case-control study	2021	Jordan	UMI	Case-control	Convenience sampling	Descriptive statistics for ranking barriers	Household survey	NR	Visually impaired	Parent	399	.	.	Toothache	.	.	.	17.9	234	165
15	Alshatrat SM	Dental Service Utilization and Barriers to Dental Care for Individuals with Autism Spectrum Disorder in Jordan: A Case-Control Study.	2020	Jordan	UMI	Case-control	Convenience sampling	Percentages	Hospital-based survey	NR	Autism Spectrum Disorder	Parent	296	192	.	Toothache	.	104		7-59	205	91
16	Alves FR	Acessibilidade Ã saÃºde bucal de pessoas com DeficiÃªncia Intelectual na perspectiva do cuidador: uma avaliaÃ§Ã£o qualitativa	2018	Brazil	UMI	Cross Sectional	Random Sampling	Mixed Method	General Public based survey	Discourse of the Collective Subject Method	Person with intellectual disability	Care givers	55	NR	NR	NR	NR	NR	NR	Mean 47.69	6	49
17	Amirian E	Preventive and curative dental services utilization among children aged 12 years and younger in Tehran, Iran, based on the Andersen behavioral model: A generalized structural equation modeling.	2025	Iran	UMI	Cross-sectional	Proportionate stratified random sampling	Structural equation modelling	Population based survey	Anderson's behavioural model	Children	Parent	886	.	.	Curative	.	507	0.572	less than 12 years (6.71 +- 3.32)	441	445
18	Anusha D	Exploring the obstacles affecting the oral health of adolescents with intellectual disabilities: insights from maternal perspectives-a qualitative study.	2025	India	LMI	Qualitative	Purposive sampling	Thematic analysis	School-based survey	NR	Adolescent with Intellectual disabilities	Mother	22	.	.	.	.	.	.	12-18	.	22
19	Bahadori M	Perceived barriers affecting access to preventive dental services: Application of DEMATEL method	2013	Iran	UMI	Cross Sectional	Systematic Random Sampling	Quantitative using DELMATE Method	Public Dental clinic	DEMATEL method	Dental Patients	Patients	100	NR	NR	Preventive Dental Care	Public based dental care	NR	NR	20 - 40	65	35
20	Bahramian H	Psychosocial determinants of dental service utilization among adults: Results from a population-based survey (Urban HEART-2) in Tehran, Iran.	2015	Iran	UMI	Cross-sectional	Multi-stage cluster random sampling	Multinomial logistic regression analysis	Community based survey (secondary data)	NR	General adults	Self-reported	20320	.	26%			8941	0.44	15-64	10160	10160
21	Bahramian H	Qualitative exploration of barriers and facilitators of dental service utilization of pregnant	2018	Iran	UMI	Qualitative	NR	Content analysis	Public health center	NR	Pregnant women	Self-reported	22	.	.	.	.	.	.	18-45 (30.4 ± 7.6)	.	22

		women: A triangulation approach.							based survey													
22	Baldani MH	Inequalities in dental services utilization among Brazilian low-income children: the role of individual determinants.	2011	Brazil	UMI	Cross-sectional	Stratified multistage sampling method	Multivariate logistic regression analysis	Household survey	Anderson's behavioural model	Children	Parent	350	.	.	.	.	.	0.31	0-14	170	180
23	Balraj L	Assessment of Oral Health Status, Care Seeking Behaviours, and Oral Health-Related Quality of Life among Indian Adults Using the World Dental Federation's Digital Application.	2024	India	LMI	Cross-sectional	Nonprobability convenience sampling	Descriptive statistics	Private dental setting	NR	General adults	Self-reported	1049	.	.	Gingivitis			0.081	38.6 ± 12.1	640	409
24	Bani Hani A	Maternal knowledge on early childhood caries and barriers to seek dental treatment in Jordan.	2021	Jordan	UMI	Cross-sectional	NR	Descriptive statistics	Health service centre	NR	Children with ECC	Mother	600	354	0.59	.	.	246	0.41	3-5	298	302
25	Barman D	Factors associated with dental visit and barriers to the utilization of dental services among tribal pregnant women in Khurda district, Bhubaneswar: A cross-sectional study.	2019	India	LMI	Cross-sectional	NR	Univariate and Multivariate logistic regression	Health service centre	NR	Pregnant women	Self-reported	300	.	.	.	.		0.155	18-37	.	300
26	Baskaradoss JK	Utilization of dental services among low and middle income pregnant, post-partum and six-month post-partum women	2020	India	LMI	Cross Sectional	Stratified random sampling	Descriptive, Chi-Square Test, ANOVA and Logistic Regression	Women and Child Hospital based survey	Andersen's Behavioral Model	Pregnant, Post Partum and 6 months post partum women	Patients	450	224	0.498	Routine Dental Check up	Public based dental care	226	0.502	18-35	NA	450
27	Benghasheer HF	ORAL HEALTH KNOWLEDGE, ATTITUDE, PRACTICE, PERCEPTIONS AND BARRIERS TO DENTAL CARE AMONG LIBYAN PARENTS.	2022	Malaysia	UMI	Cross-sectional	Nonprobability purposive sampling	Percentages	School-based survey	NR	Children	Parent	381	.	.	.	.	.	.	1- 7	.	.
28	Benjamin N	Oral Healthcare Utilization Factors Shaping the Perceived Oral Health Outcome Among Gond Tribes of Chhattisgarh: A Cross-Sectional Study Based on Andersen's Behavioral Model.	2024	India	LMI	Cross-sectional	NR	Binary logistic regression analysis	Household survey	NR	General adults	Self-reported	400	.	.	Curative	.	.	.	15-55 (34.84 ± 12.2)	191	209
29	Bhaskar BV	Self-perception on oral health and related behaviours among	2020	India	LMI	Cross Sectional	Convenience Sampling	Descriptive and Chi-Square Test	Government antenatal	NR	Antenatal Women	Patients	400	53	0.132	Emergency	Government dental	119	0.298	Mean 27	NA	400

		antenatal mothers attending a public antenatal clinic â€“ Kerala							clinic based survey							and Need Based Dental Care	services					
30	Bhaskar BV	Access to dental care among differently-abled children in Kochi	2016	India	LMI	Cross Sectional	Convenience Sampling	Descriptive and Chi-Square Test	School based (Special and integrated schools)	Penchansky & Thomas Access Model	Differently abled schoolchildren	Care givers/Patients	331	65	0.196	Emergency and Need Based Dental Care		224	0.676	6-14	214	117
31	Bhat N	Barriers to restorative care as perceived by patients attending government hospitals in Udaipur, Rajasthan	2014	India	LMI	Cross Sectional	Convenience Sampling	Descriptive, Chi-Square Test and ANOVA	Government Hospital	Behavioral Focus	Dental Patients	Patients	242	148	0.612	Curative Dental Care	Government dental services	94	0.388	21-60	163	79
32	Bhatt S	Dental Caries Experience and Utilization of Oral Health Services Among Tibetan Refugee-Background Children in Paonta Sahib, Himachal Pradesh, India	2018	India	LMI	Cross Sectional	Convenience Sampling	Descriptive, t-Test and Chi-Square	Sambhota Tibetan School, refugee settlement	NR	Tibetan Refugee Schoolchildren	Care givers	254	100	0.394	Curative Dental Care (extraction-43%, filling-32%)	NR	154	0.606	6-18	124	130
33	Bhatt S	Factors influencing oral health and utilization of oral health care in an Indian fishing community, Mangaluru city, India	2017	India	LMI	Cross-sectional	Cluster sampling	Descriptive statistics for ranking barriers and Chi-square for association	Household survey	NR	General adults	Self-reported	840	.	.	Extraction (69.2%)	.	463	0.551	18-59 (median 38.5)	469	371
34	Bhuvaneshwari NG	Perceived Dental Needs and Barriers to Utilization of Dental Services Among Elders in India â€“ A Cross-Sectional Survey	2021	India	LMI	Cross Sectional	Simple random sampling	Quantitative with structured questionnaire	Community-based (households and shelter homes)	Andersen's Behavioral Model	Elderly	Patients	1440	416	0.705	Curative Dental Care	Government dental services	424	0.295	Mean 68.1	921	519

35	Bommireddy VS	Oral hygiene habits, oral health status, and oral health care seeking behaviors among spinning mill workers in Guntur district: A cross-sectional study	2020	India	LMI	Cross Sectional	Simple random sampling	Quantitative with structured questionnaire	Spinning mills in and around Guntur district, Andhra Pradesh	NR	Industrial workers (spinning mill workers)	Patients	458	144	0.315	Curative Dental Care	Government dental services	314	0.6856	18-51	229	222
36	Bommireddy VS	Dental Service Utilization: Patterns and Barriers among Rural Elderly in Guntur District, Andhra Pradesh.	2016	India	LMI	Cross-sectional	Stratified cluster sampling	Descriptive statistics for ranking barriers and Chi-square for association	Rural Household survey	NR	Older adults (>55)	Self-reported	621		0.319	Extraction	Dental institution, Private services	424		55-86 (64±7.68)	345	276
37	Bommireddy VS	Socio-economic Status, Needs, and Utilization of Dental Services among Rural Adults in a Primary Health Center Area in Southern India.	2014	India	LMI	Cross-sectional	Cluster sampling	Percentages	Rural Household survey	NR	General adults	Self-reported	385	.	.	Extraction	.	57	0.3115	35-44	190	195
38	Braimoh OB	Utilisation of Dental Services Among Patients in a Tertiary Health Institution in Nigeria Contact Author	2013	Nigeria	LMI	Cross Sectional	Convenience Sampling	Quantitative with structured questionnaire	Oral diagnosis unit, University of Benin Teaching Hospital	NR	General adult dental patient	Patients	390	288	0.738	Curative Dental Care	Government dental services	NR	NR	Mean 25-34	149	241
39	Bunnatee P	Factors associated with oral health care behaviors of pregnant women in a northeastern province in Thailand: A hospitalbased cross-sectional study	2023	Thailand	UMI	Cross Sectional	Convenience Sampling	Quantitative with structured questionnaire	Antenatal care clinics of government hospitals	Instrument adopted from HeLD-Th (Health Literacy in Dentistry)	Pregnant women attending antenatal care	Patients	405	217	0.536	Curative Dental Care		188	0.464	Mean 26.3	NA	405
40	Cai Y	Factors associated with oral health service utilization among young people in southern China.	2024	China	UMI	Cross-sectional	Stratified random sampling	logistic regression	University survey	NR	College students	Self-reported	662		0.495	Curative (59.6%), Preventiv	.	.	.	17-25	327	335

																e (12.8 %)						
41	Chandu VC	Is the Intention Being Realized in Execution: Evaluation of Oral Health Promotion Program at a South Indian University	2019	India	LMI	Cross Sectional	Two-Stage Random sampling	Quantitative with structured questionnaire	University based survey	NR	University students and staff	Patients	301	28	0.093	Emergency and Need Based Dental Care	NR	273	0.907	16-25	143	158
42	Condessa AM	Use of dental services by disability status in Brazil in 2013	2021	Brazil	UMI	Cross-sectional	Cluster sampling	Bivariate and Multivariate logistic regression analysis	Nationwide household survey (secondary data)	modified Andersen and Davidson model	Disabled adults	Self-reported	57201		0.341	.	.			Mean 55.4	.	.
43	Cordeiro D	Utilization of dental services by rural riverside populations covered by a Fluvial Family Health Team in Brazil	2024	Brazil	UMI	Cross Sectional	Stratified random sampling	Quantitative with structured questionnaire	Rural riverside localities on the left bank of the Rio Negro, Manaus, Amazonas, Brazil	Andersen's Behavioral Model	Rural riverside localities adults	Patients	492	250	0.508	Curative and Preventive Dental Care	Government Hospital	15	0.031	Mean 43.5	240	252
44	Cossio-Alva BA	Factors associated with the use of dental services in older adults in Peru.	2025	Peru	UMI	Cross-sectional	probabilistic stratified and multi-stage sampling	Multivariate analysis	Nationwide household survey (secondary data)	NR	Older adults	Self-reported	30318			.	.	4540	0.15	60-90 (most: 60-69)	14329	15988
45	Cruz G	Factors associated with the use of dental health services in primary care in northeastern Mexico	2016	Mexico	UMI	Cross-sectional	NR	Descriptive statistics	Household survey	NR	Students	Parent	92		0.533	.	Welfare brigade			11.3 ± 7.7	39	53
	Cruz G										Pregnant women	Self-reported	92		0.467	.	Social security/popular insurance			25.1±6.0	.	92

	Cruz G										Active workers	Self-reported	92		0.402	.	Social security/popular insurance			32.8±8.6	61	31
	Cruz G										Older adults (>60)	Self-reported	92		0.435	.	Social security/popular insurance			66.7±8.0	36	56
46	Curi DSC	Utilization of oral health services network among children and adolescents with leukemia.	2018	Brazil	UMI	Cross-sectional	NR	Descriptive statistics	Health service centre	Anderson's behavioural model	Leukemia patients	Care giver	69	29	0.547	Curative	Public services (85.5%)	.	.	3–16	36	33
47	da Silva AN	The association between low-socioeconomic status mother's Sense of Coherence and their child's utilization of dental care	2011	Brazil	UMI	Cross-sectional	NR	Multiple logistic regression	School-based survey	Anders en and Davidson 1997	Children	Mother	190	154	0.811	Treatment (57.1%), Dental check-up (42.9%)	Public services (54.5%), Private services (33.1%)	36	0.189	11.6 ± 0.95	83	107
48	da Silva-Selva ELMS	Oral Health Care of Children and Adolescents with Different Impairments of Cerebral Palsy: Barriers and Challenges	2022	Brazil	UMI	Cross -sectional	NR	Descriptive statistics	Health service centre	NR	Cerebral Palsy	Care giver	94	86	0.915	Urgency/treatment, Revisit	Public service (64.7%), Private service (35.3%)	8	0.085	5-18	61	33
49	Dahal S	Utilization of oral health care services and perceived barriers among adults residing in Jiri, Nepal: a cross-sectional study	2025	Nepal	LMI	Cross Sectional	Convenience Sampling	Quantitative with structured questionnaire	Rural community (Jiri municipality, Dolakha district, Bagmati Province, Nepal)	NR	Adult residents of Jiri municipality	Patients	725	130	0.179	Emergency and Need Based Dental Care	Government Hospital	595	82.1	Mean 48.78 ± 15.30	375	350
50	Daou D	Prevalence and socioeconomic factors associated with non-utilization of dental care in Lebanon: A nationwide cross-sectional survey.	2024	Lebanon	UMI	Cross-sectional	Probabilistic stratified sampling	Univariate and Bivariate Logistic regression	Nationwide household survey (secondary data)	NR	General adults	Self-reported	1070	970	0.907	Pain/emergency (53%),	.	144	0.135	18-88	643	427

																Routine visit (23.6%)						
51	de Albuquerque OM	[Pregnant women's perceptions of the Family Health Program concerning barriers to dental care in Pernambuco, Brazil].	2004	Brazil	UMI	Qualitative	NR	Content analysis	Focus group discussion	NR	Pregnant women	Self-reported	.	.	.	.	.	.	.	.	.	.
52	de Gutierrez GM	Barriers to access to dental treatment for people with physical disabilities in a Brazilian metropolis	2018	Brazil	UMI	Cross-sectional	NR	Percentages	Health service centre	NR	Physical disability	Care giver	191	.	.	.	.	78	0.408	6.2 ± 4.5	112	79
53	de Matos M	Perception of elderly women with depressive symptoms regarding access to oral healthcare in the Family Health Strategy	2024	Brazil	UMI	Qualitative	Random	Content Analysis	Household survey	NR	Older adults	Self-reported	20	.	0.65	.	.	.	.	60-84	.	20
54	de Souza MLP	Utilization of dental services by children with autism spectrum conditions: The role of primary health care.	2024	Brazil	UMI	Cross-sectional	NR	Bivariate and Multivariate logistic regression analysis	Health service centre	NR	Children with Autism Spectrum Disorder	Care giver	100	57	0.57	.	.	25	0.25	06-12	6	94
55	Deghatipour M	Oral health status in relation to socioeconomic and behavioral factors among pregnant women: A community-based cross-sectional study	2019	Iran	UMI	Cross Sectional	Convenience Sampling	Quantitative with structured questionnaire	Public maternal healthcare centers in Varamin region, southern Tehran Province	NR	Pregnant women in 2nd and 3rd trimesters	Patients	407	205	0.504	Emergency and Need Based Dental Care	Government Hospital	202	49.6	15-44	NA	407
56	Denloye O	Dental service utilization among junior secondary school students in Ibadan, Nigeria	2010	Nigeria	LMI	Cross Sectional	Multi Stage random Sampling	Quantitative with structured questionnaire	School based Survey	NR	School going students	Patients	457	63	0.138	Emergency and Need Based Dental Care	NR	392	85.8	8-16	217	240

57	Deolia SG	Evaluation of oral health care seeking behavior in rural population of central India	2020	India	LMI	Cross Sectional	Convenience Sampling	Quantitative with structured questionnaire	Rural community based survey	Oral Health literacy and Health seeking behavior construct	Rural adult population	Patients	700	372	53.1	Emergency and Need Based Dental Care	Government Hospital	328	46.9	21-40	353	347
58	Devaraj C	Reasons for use and non-use of dental services among people visiting a dental college hospital in India: A descriptive cross-sectional study.	2012	India	LMI	Cross-sectional	Deliberate sampling	Percentages	Health service centre	NR	General adults	Self-reported	180	122		Dental pain (35.3%)	.	58		15-65		
59	Eigbobo JO	Utilization of dental services among secondary school students in Port Harcourt, Nigeria	2016	Nigeria	LMI	Cross-sectional	Convenience sampling	Descriptive statistics	School-based survey	NR	Children	Self-reported	200	70	0.35	.	.	130	0.35	12-15 (13.3 ±1.1)	98	102
60	Fakheran O	The impact of pregnancy on women's oral health-related quality of life: a qualitative investigation	2020	Iran	UMI	Qualitative Descriptive Study	Purposive sampling	Interpretative Phenomenological Analysis (IPA)	Public healthcare centers in Isfahan city	WHO Quality of Life Framework (WHOQOL)	Pregnant women	Patients	27	NR(qualitative study)	NR	Emergency and Need Based Dental Care	NR		NA	17-41	NA	27
61	Falquez M	Association between type of health insurance and dental visits among Ecuadorian older population: evidence from a cross-sectional study.	2025	Ecuador	UMI	Cross-sectional	Probabilistic sampling	Logistic regression	Nationwide survey (secondary data)	NR	Older adults	Self-reported	4565	.	.	.	.	.	.	>60	2215	2350
62	Fayyaz F	Pattern of dental health status among sensory impaired children of district Lahore: Influence of parental socioeconomic status	2021	Pakistan	LMI	Cross-sectional	NR	Descriptive statistics	School-based survey	NR	Sensory Impaired Children	School instructors	250	124	0.496	.	.	188	0.752	9-13	171	79
63	Ferreira C	Factors associated with the use of dental services by elderly Brazilians	2014	Brazil	UMI	Cross-sectional	Multistage sampling	Poisson regression	Nationwide household survey (secondary data from SB	NR	Older adults	Self-reported	6702	3118	0.465	Treatment, Prosthetic use	.	.	.	65-74	2582	4120

									Brasil 2010)													
64	Folorunsho S	Access to and Utilization of Dental Care Services by Older Adults in Nigeria: Barriers and Facilitators	2025	Nigeria	LMI	Qualitative Descriptive Study	Purposive sampling	Inductive thematic analysis (Braun & Clarke)	Urban and peri-urban community based study	Inductive grounded approach	Older Adults	Patients	15	9	0.6	Emergency and Need Based Dental Care	Teaching Hospital/Public Hospital dental unit, Urban centre	6	0.4	50-83	9	6
65	Fonseca SGOD	Factors associated with public dental service use by adults in the state of SÃ£o Paulo, Brazil, 2016.	2020	Brazil	UMI	Cross-sectional	Two-stage cluster sampling	Logistic regression	Community based survey (secondary data)	Anderson's behavioural model	General adults	Self-reported	5709	3162	0.561	Curative/pain (75.0%), Check-up (25.0%)	Private services (59.9%),Public services (40.1%)	.	.	35-45	1829	3880
66	Fotedar S	Barriers to the utilization of dental services in Shimla, India	2013	India	LMI	Cross-sectional	Random sampling method	Quantitative with structured questionnaire	Rural household based study	NR	Adults	Patients	304	46	0.151	Emergency and Need Based Dental Care	NR	159	0.523	25-45	162	142
67	Frusca do Monte CM	Access to dental care for patients with inherited bleeding and hemoglobin disorders.	2022	Brazil	UMI	Cross-sectional	NR	Poisson regression	Health service centre	NR	Inherited bleeding and Hemoglobin disorders	Parent(children) and self-reported(adults)	131	76	0.58	Preventive treatment (53.2%) Curative treatment (46.8%)	Private service (41%), Public service (38.5%), Specialised Hematology center	.	.	1-85 (22.6 ± 19.06)	93	38

																	(20.5%)					
68	Galicia-Diez Barroso D	Using the Expanded Andersen Model to Determine Factors Associated with Mexican Adolescents' Utilization of Dental Services.	2023	Mexico	UMI	Cross-sectional	Non-probabilistic sampling	Logistic regression	School-based survey	expanded Andersen model	Adolescents	Self-reported	247	125	0.5061	Check-ups and cleaning (56%), Pain (23.20%) , Restorative treatment (15.20%)	Public services (28.80%), Private services (56%) , Teaching clinics and pharmacies’ (15.20%)	.	.	13.51 ± 1.09	119	128
69	GalvÃ£o MHR	Using Andersen's behavioural model to examine individual and contextual factors associated with dental service utilization in Brazil.	2023	Brazil	UMI	Cross-sectional	Cluster sampling	Multinomial logistic regression analysis	Nationwide household survey (secondary data from Brazilian 2019 National Health Survey)	Anderson’s model 2013	General adults	Self-reported	94114	.	0.497	.	.	.	0.02	>15	.	.
70	Gao X	Utilization of dental services and associated factors among preschool children in China	2020	China	UMI	Cross -sectional	Multi Stage random Sampling	Quantitative with structured questionnaire	Nationwide Urban and rural community based survey	Andersen’s Behavioural Model	Preschool children	Care giver/Parents	40305	5281	0.131	Curative dental care	NR	33211	0.824	3-5	20226	20070
71	Garcha V	Barriers to oral health care amongst different social classes in India.	2010	India	LMI	Cross-sectional	Convenience sampling	Percentages	Household survey	NR	General adults	Self-reported	50	.	.	.	.	.	.	35-45 (39.7± 3.2)	156	94
72	Gomes A	Fatores associados ao uso dos serviÃ§os de saÃºde bucal: Estudo de base populacional em	2014	Brazil	UMI	Cross-sectional	Two-Stage Stratified	Quantitative Survey Based	Urban community based survey	Andersen’s Behavi	Children and Adults	Care giver/Parents	13059	402	Children-8.6% ,	Emergency and	Children- SUS, Adults	1868	Children -	Children - 0-5, Adults - 20-59	.	.

		municÃpios do MaranhÃ£o, Brasil					Cluster sampling			oural Model					Adults - 28.1 %	Need Based Dental Care	- Private		91 %, Adults - 71.9%			
73	GONÃ‡ALVES, I da Costa	Utilization of dental services by preschool children: prevalence and associated factors	2024	Brazil	UMI	Cross-sectional	Random Sampling	Quantitative Survey Based	Public Health Service centre	Andersen's Behavioural Model	Pre-Schoolchildren	Care giver	308	122	0.396	Curative dental care	Public Services UHS/ BHU	186	0.604	1-3	139	169
74	Gopika GG	Oral Health Challenges and Barriers to Dental Care Access Among Narikuravar Gypsies in Chennai: A Cross-Sectional Study	2025	India	LMI	Cross-sectional	NR (Census approach)	Quantitative with structured questionnaire	Community-based field survey in an urban marginalized tribal population	NR	Marginalized tribal adults	Patients	460	NR	NR	NR	NR	NR	NR	1-70	253	197
75	Gupta N	Knowledge and Practices of Pregnant Women regarding Oral Health in a Tertiary Care Hospital in Nepal.	2019	Nepal	LMI	Qualitative	Convenience sampling	Content analysis	Health service centre	NR	Pregnant women	Self-reported	50	.	.	.	.	.	.	17-38 (25.12 ± 5.4)	.	50
76	Gupta S	Oral health services utilization among the rural population of western Rajasthan, India	2014	India	LMI	Cross-sectional	Convenience Sampling	Quantitative with structured questionnaire	Rural Satellite dental OPD based survey	NR	Adults in rural population	Patients	5476	82	0.0149	Emergency and Need Based Dental Care	Public satellite dental OPDs	3004	0.5485	20-60	3934	1542
77	Harirugsakul P	Social backgrounds, oral behaviors and dental service utilization among Thai older adults: data from the national oral health survey	2020	Thailand	UMI	Cross-sectional	Stratified multi--stage random sampling	Quantitative with structured face to face interviews	Nationwide Urban and rural community based survey	Social determinant frame work	Older Adults	Patients	4130	1569	0.38	Emergency and Need Based Dent	Government dental services	NR	NR	60-74	2001	2129

																al Care						
78	Hegde AM	SPECIAL NEEDS OF SPECIAL CHILDREN-PARENTAL VIEW	2015	India	LMI	Cross-sectional	Purposive sampling	Quantitative with structured questionnaire	School based Survey	NR	Children with disabilities	Care giver/Parents	250	28	0.11	Emergency and Need Based Dental Care	Private dental services	NR	NR	NR	NR	NR
79	Hemani A	Barriers to the access of oral health care in individuals from lower socioeconomic communities in Karachi	2017	Pakistan	LMI	Cross-sectional	NR	Descriptive statistics	Health service centre	NR	General adults	Self-reported	385	.	0.46	.	.		0.538	26-65	192	193
80	Herkrath FJ	Utilisation of dental services by Brazilian adults in rural and urban areas: a multi-group structural equation analysis using the Andersen behavioural model.	2020	Brazil	UMI	Cross-sectional	Cluster sampling	Confirmatory factor analysis (CFA) and Multi-group structural equation modeling	Nationwide survey (secondary data)	Anderson's behavioural model	General adults	Self-reported	60202	.	0.444	.	.	.	0.033	42.9	28355	31847
81	Herkrath FJ	Contextual and individual factors associated with dental services utilisation by Brazilian adults: A multilevel analysis.	2018	Brazil	UMI	Cross-sectional	Probabilistic simple random sampling	Multilevel logistic regression	Nationwide survey (secondary data)	Anderson's behavioural model	General adults	Self-reported	27017	.	0.481	.	.	.	0.018	18-65	12158	14859
82	Horn R	Perceptions of oral health among older Cambodians and their caregivers: A qualitative study.	2018	Cambodia	LMI	Qualitative	Convenience sampling	Thematic analysis	Focus group interviews	NR	Older adults	Self-reported	56	.	.	.	.	.	.	.	20	36
83	Jadhav SK	Patterns, barriers, and utilization of oral healthcare provided at primary health centers (PHCs) and its association with oral health status among rural population in Rohtak district, Haryana: A household cross-sectional study.	2024	India	LMI	Cross-sectional	Multistage cluster systematic random sampling	Descriptive statistics	Household survey	NR	General adults	Self-reported	600			.	Private dentist (55.8%), Primary Health centre (21.3%)				223	377
84	Jadoon AH	Experiences of transwomen individuals in accessing oral health care in a low and middle-	2025	Pakistan	LMI	Qualitative	Purposive sampling	Thematic analysis	Community based survey	NR	Transwomen	Self-reported	10	.	.	.	.	.	.	.	.	.

		income country: a qualitative study from Pakistan.																				
85	Jaidee J	Prevalence and Factors Associated with the Utilization of Dental Care Services among Factory Workers in Nava Nakorn Industrial Estate, Pathumthani Province, Thailand.	2015	Thailand	UMI	Cross-sectional	Probability sampling	Logitic regression	Community based survey	NR	General adults	Self-reported	1500	541	0.361	Pain, tooth decay, routine check-ups	Private service	959	0.639	19-59 (31.75)		
86	Jain V	" Barriers in Utilization of Oral Health Care Services Among Patients Attending Primary and Community Health Centres in Virajpet, South Karnataka'' "Barriers in Utilization of Oral Health Care Services Among Patients Attending Primary and Community Health Centres in Virajpet, South Karnataka''	2013	India	LMI	Cross-sectional	Multi Stage random Sampling	Quantitative with structured questionnaire	Rural PHCs and CHCs	Andersen's Behavioral Model	Adults	Patients	600	62	0.1035	Emergency and Need Based Dental Care	Government based PHC and CHCs dental services	NR	NR	20-50	280	320
87	James A	Perception of Caregivers about Oral Health Services for Institutionalized Older Adults: A Mixed Method Study.	2024	India	LMI	Mixed Methods	Convenience sampling	Thematic analysis	Old age homes	NR	Older adults	Care giver	54	.	.	.	.	.	.	>60	13	41
88	Jena S	The Barrier to Accessing Dental Healthcare Services Among the Institutionalized Visually Impaired Adults: A Qualitative Study.	2024	India	LMI	Qualitative	NR	Content analysis	Special care home	NR	Visually impaired	Self-reported	20	.	.	.	.	.	.	37 ± 16.79	9	11
89	Jessani A	Oral Health Status and Patterns of Dental Service Utilization of Adolescents in Lesotho, Southern Africa.	2021	Lesotho	LMI	Cross-sectional	Convenience sampling	Univariate and multivariable logistic regression	School-based survey	Andersen & Newman Health Services Utilization Model	Adolescents	Self-reported	526	60	0.116	Emergency/pain	Public health clinics/ hospitals	363	0.702	12-19 (16.4)	164	355
90	Joseph C	Utilization of Dental Services by Rural dwellers: Case of two Communities hosting a tertiary health facility	2022	Nigeria	LMI	Descriptive Cross-	Systematic Househol	Quantitative with structured questionnaire	Rural community based survey	NR	Adults	Patients	278	58	0.209	Need Based	UNTH Dental Service	220	0.791	18-80	106	172

						sectional	d Sampling									and Preventive Dental Care						
91	Joudi A	To appreciate the influence of contributed determinants on dental care utilization in the context ofÂ socio-economic inequalities.	2024	Iran	UMI	Cross-sectional	Proportionate stratified random sampling	Poisson regression	Community based survey	NR	General adults	Self-reported	1510	.	.	.	.	.	.	18-65 (46.21 ± 18.43)	753	757
92	Kachwinya SM	Oral health status and barriers to oral healthcare among children with cerebral palsy attending a health care center in Kampala, Uganda.	2022	Uganda	LI	Cross-sectional	Convenience sampling	Descriptive statistics	Health service centre	NR	Children with cerebral palsy	Care giver	90	.	.	Emergency treatment (swelling/ pain/ mobile teeth)	.	.	.	3-17	45	45
93	Kadaluru UG	Utilization of oral health care services among adults attending community outreach programs.	2012	India	LMI	Cross-sectional	NR	Percentages	Health service centre	NR	General adults	Self-reported	246	69	0.28	Extraction of tooth (11%), restorative care (6%)				34.5 ± 9.66	30	216
94	Kakatkar G	Barriers to the utilization of dental services in udaipur, India.	2011	India	LMI	Cross-sectional	Simple random sampling	Spearman's correlation	Household survey	Andersen's Model	General adults	Self-reported	427		0.04					25-45	248	179
95	Karam I	Barriers to the use of dental services by children in Lebanon and association with parental perception of oral health care	2020	Lebanon	UMI	Cross-sectional	Convenience sampling	Logistic regression	School-based survey	NR	Children	Parent	316	.	0.613	Decay and acute pain (89.6%), Regular check-up (66.5%)	.	.	.	7-12 (9.5 ± 1.5)	.	.

96	Khairunjauhari IM	Oral Health Beliefs, Perceptions and Utilisation of Oral Health Care Services among the Indigenous People (Orang Asli) in Pahang, Malaysia: A Qualitative Study.	2023	Malaysia	UMI	Qualitative	Snowball sampling	Thematic analysis	Focus group discussion	NR	General adults	Self-reported	19	.	.	.	.	.	.	18-46	9	10
97	Khuong AP	The Barriers in Using Oral Health Services of People Aged 18 or Over in Danang in 2023: A Cross-sectional Study.	2024	Vietnam	LMI	Cross-sectional	Convenience and purposive sampling	Univariate and Multivariate logistic regression	Community based survey	Jean-Frederic Levesque's access model	General adults	Self-reported	386		0.425				0.575	18–39 (35.47 ± 14.81)	145	241
98	Kikwilu EN	Prevalence of oral pain and barriers to use of emergency oral care facilities among adult Tanzanians	2008	Tanzania	LMI	Cross-sectional	Purposive Cluster sampling	Quantitative structured household interviews with pretested questionnaire	Urban and rural household community based survey	Andersen's Behavioral Model	Adults	Patients	1759	741	0.421	Emergency and Need Based Dental Care	Government Dental Clinic in hospital	1018	0.579	18-92	864	895
99	Kikwilu EN	Barriers to restorative care as perceived by dental patients attending government hospitals in Tanzania.	2009	Tanzania	LMI	Cross-sectional	Stratified sampling	Descriptive statistics	Health service centre	NR	General adults	Self-reported	1138	.	.	.	.	.	.	15-50	.	.
100	King T	Tooth brushing and utilization of dental services in Fiji (1998).	2003	Fiji	UMI	Cross-sectional	Stratified multistage cluster sampling	Percentages	NR	NR	General adults	Self-reported	619	.	.	.	.	.	.	15-44	.	.
101	Krishnan L	Factors affecting the unmet dental needs and dental service utilisation among urban slum dwellers of Chennai city, India	2019	India	LMI	Descriptive Cross-sectional	Multi-Stage random sampling	Quantitative face to face structured questionnaire	Urban slum communities	Andersen's Behavioral Model	Urban slum dwellers	Patients	400	25	0.063	Emergency and Need Based Dental Care	Government Hospital	253	0.632	18-80%	175	225
102	Krishnan L	Barriers in dental care delivery for children with special needs in chennai, india: A mixed method research	2018	India	LMI	Mixed Methods	Convenience sampling	Thematic analysis	Health service centre	NR	Children with Special Needs	Parent/Caregiver	100	.	0.446	.	.	.	.	3-15	.	100

103	Kumar G	Barriers in Dental Care Utilization-An Explorative Study among Transgender Community of Bhubaneswar, Odisha.	2023	India	LMI	Qualitative	NR	Thematic analysis	Special home	NR	Transgender	Self-reported	150	.	.	.	.	.	.	24-44	.	.
104	Lalitha ND	Accessibility and barriers to oral health care among gypsy tribes in Chennai: A cross sectional study	2019	India	LMI	Cross-sectional	Convenience sampling	Descriptive statistics	Community based survey	NR	General adults (Gypsy tribes)	Self-reported	102	32	0.313	Decay and pain	Private services (72.60%), Public services (21.60%)	26	0.254	12-70	34	68
105	Leal Rocha L	Access to dental public services by disabled persons.	2015	Brazil	UMI	Cross-sectional	Probabilistic sampling	Descriptive statistics	Household survey	NR	People with Disabilities	Self-reported/ Care giver	204	97	0.475	Emergency care (pain) (84.5%)	Primarily at public FHU; 23.5% used private services		0.4	3-97 (39.8 ± 22.7)	112	92
106	Li C	Disparities in dental healthcare utilization in China.	2018	China	UMI	Longitudinal	Multistage probability-based sampling	Concentration index and regressions	Nationwide survey (secondary data)	NR	General adults	Self-reported	17648	2989	0.169	.	Private clinics	.	.	>45	8548	9100
	Li C	Year 2013, 2015											15450	2791	0.181	.	.	.	.		7403	8047
107	Luksamijarulkul N	Awareness and attitude towards dental coverage right affecting the dental service utilization among selected thais in Bangkok.	2024	Thailand	UMI	Cross-sectional	Convenience sampling	Multivariate analysis	Community based survey	NR	General adults	Self-reported	315	229	0.7269	.	.	84	0.2666	15-60	145	168
108	Machiri, S.	Ecological risk factors for unmet needs in oral health care in Chegutu rural district in Zimbabwe a cross-sectional study	2021	Zimbabwe	LMI	Cross-sectional	Systematic random sampling	Logistic regression	Health service centre	NR	General adults	Self-reported	276		0.109	.	.	.	.	median : 39.5	100	176
109	Machry, RÃ´mulo V.	Socioeconomic and psychosocial predictors of dental healthcare use among Brazilian preschool children	2013	Brazil	UMI	Cross-sectional	Stratified cluster sampling	Quantitative with structured questionnaire	Public health centers	Andersen's Behavioral Model	Preschool children	Care giver /Parents	478	112	0.236	Emergency and Need Based	More Public Sectors	361	0.7632	Mean 34	232	246

																Dental Care						
110	Maffioletti F	Predisposing, enabling, and need characteristics of dental services utilization among socially deprived schoolchildren.	2020	Brazil	UMI	Cross-sectional	NR	Structural equation modeling (SEM) and Confirmatory factorial analysis (CFA)	Household survey	Anderson's behavioural model	Children	Parent	358	171	0.478	Prevention or check-up (31%), Curative (26%)		106	0.296	12	149	209
111	Maharani DA	Inequity in dental care utilization in the Indonesian population with a self-assessed need for dental treatment.	2009	Indonesia	UMI	Cross-sectional	Cluster sampling	Concentration index and regressions	Nationwide Socio-Economic Survey	NR	General adults	Self-reported	20718	7155	0.345	.	.			>15	10103	10615
112	Malhi R	Perceived barriers in accessing dental care among patients attending dental institute using decision-making trial and evaluation laboratory method	2015	India	LMI	Cross-sectional	Convenience Sampling	Quantitative with structured questionnaire	Dental college outpatient department	DEMATEL Frame work	Adults	Patients	364	NR	NR	Emergency and Need Based Dental Care	Private dental college hospital	NR	NR	20-30	201	163
113	Marlecha RB	Oral health status, dental awareness, and dental services utilization barriers among transgender population in Chennai	2020	India	LMI	Cross-sectional	Convenience Sampling	Quantitative with structured questionnaire	Urban, community- NGO based survey	NR	Transgender individual	Patients	72	29	0.4	Emergency and Need Based Dental Care	Private or general dental hospitals	43	0.6	Mean 34.2 ± 14.1	NA	NA
114	Martins AM	[Utilization of dental services among the elderly in Brazil].	2007	Brazil	UMI	Cross-sectional	Cluster sampling	Univariate and Multivariate analysis	Nationwide household survey (secondary data)	Andersen and Davidson 1997	Older adults	Self-reported	5349	4114	0.774	.	.	310	0.058	65-74	1920	3089
115	Masiga MA	Navigating the healthcare system in Nairobi City County: perspectives and experiences in the utilization of oral healthcare	2022	Kenya	LMI	Mixed Methods	Purposive sampling	Chi square/ANOVA for quantitative and content	Health service centre	NR	Children with HIV/AIDS	Care giver	221	.	0.19	Emergencies	Private clinics (57.1%), Public	.	.	>18	.	221

		by caregivers of children with HIV/AIDS.						analysis for qualitative									services (19.5%)					
116	Matos DL	The BambuÃ Project: a population-based study of factors associated with regular dental care in adults Palavras-chave Odontologia; AssistÃªncia OdontolÃ³gica; SaÃºde do Adulto	2001	Brazil	UMI	Cross-sectional	Simple random sampling	Quantitative structured household interviews using validated questionnaire	Urban community based survey	Andersen and Newman Model	Adults	Patients	645	161	0.246	Emergency and Need Based Dental Care	Private or general dental hospitals	NR	NR	18-89	316	338
117	Matos DL	Socio-demographic factors associated with dental services among Brazilian older adults: a study based on the National Household Sample	2004	Brazil	UMI	Cross-sectional	Stratified multi--stage sampling	Quantitative – secondary data analysis from PNAD using structured interviews	Community - based household survey	Andersen's Behavioral Model	Older Adults	Patients	28943	3820	0.132	Emergency and Need Based Dental Care	Public Dental Services	1823	0.063	majority- 60-64	12771	16172
118	Medina-SolÃs CE	Factors Inï¬‚uencing the Use of Dental Health Services by Preschool Children in Mexico	2006	Mexico	UMI	Cross-sectional	Census of 10 public preschool enrolled	Quantitative with structured questionnaire	School based Survey	Andersen's Behavioral Model	Preschool children	Caregivers/Patients	1303	403	0.31	Curative based dental care	Private dental services	883	0.678	3-6	673	630
119	Medina-SolÃs CE	Clinical and non-clinical variables associated with preventive and curative dental service utilisation: A cross-sectional study among adolescents and young adults in Central Mexico	2019	Mexico	UMI	Cross-sectional	Random Sampling	Quantitative Multinominal logistic regression	University based survey	Andersen's health service uthilization Model	Adolescent and young adults	Patients	638	407	0.638	Curative based dental care	NR	10	1.6	Mean 18.76 ± 1.76	324	314
120	Miranda CD	[Determinants of dental services utilization by adults: a population-based study in FlorianÃ³polis, Santa Catarina State, Brazil].	2013	Brazil	UMI	Cross-sectional	NR	Poisson regression	Community based survey	NR	General adults	Self-reported	1720	1136	0.666	.	Private service (76.3%), Public (18.9%)	7	0.006	20-59	761	959

121	Mohamadi-Bolbanabad, A.	Unmet dental care need in West of Iran: determinants and inequality	2021	Iran	UMI	Cross-sectional	Multistage sampling	Multivariate logistic regression and Concentration index	Health service centre	NR	General adults	Self-reported	1056	.	0.393	Treatment (Restoration, Extraction)	.	.	.	>18	537	519
122	Mohanty V	Oral healthcare-related perception, utilization, and barriers among schoolteachers: A qualitative study.	2021	India	LMI	Qualitative	Purposive sampling	Summative content analysis	School-based survey	NR	General adults	Self-reported	44	.	.	.	.	.	.	27-41 (33.7 ± 7.02)	3	41
123	Mohapatra S	ASSESSMENT OF ORAL HEALTH STATUS, SELF-PERCEIVED NEEDS, UNMET NEEDS, AND BARRIERS TO UTILIZATION OF DENTAL SERVICES AMONG INSTITUTIONALISED ELDERLY POPULATION IN CHENNAI, INDIA: A CROSS SECTIONAL STUDY	2023	India	LMI	Cross-sectional	Stratified random sampling	Descriptive statistics	Elderly home	NR	Older adults	Self-reported	334	.	.	.	.	.	.	65-74 (68.61)	131	203
124	Mohd FN	Perceptions Toward Healthcare and Dental Care Services among Parents and Caretakers of People with Intellectual Disability (PWID)-A Questionnaire Study.	2023	Malaysia	UMI	Cross-sectional	Purposive sampling	Percentages	Community centre survey	NR	People with intellectual disabilities	Parent/Caregiver	189	104	0.55	.	.	44	0.233	43.96 ± 12.1	40	149
125	Molete MP	Oral health needs and barriers to accessing care among the elderly in Johannesburg.	2014	South Africa	UMI	Cross-sectional	Stratified sampling	Logistic regression	Household survey	NR	Older adults	Self-reported	308	85	0.276	.	.	223	0.724	>60	107	201
126	Momeni Z	Perceived barriers to the preservation and improvement of children's oral health among Iranian women: a qualitative study.	2019	Iran	UMI	Qualitative	Purposive sampling	Content Analysis	Focus group discussion	NR	Children	Mother	58	.	.	.	.	.	.	.	.	58
127	Monteiro CN	Socioeconomic inequalities in dental health services in Sao Paulo, Brazil, 2003-2008.	2016	Brazil	UMI	Cross-sectional	Probability sampling	Logistic regression	Household survey (secondary data)	NR	General adults	Self-reported	1667	611	0.467	.	.	.	.	>20	803	864
	Monteiro CN	Year 2003, 2008											2086	1002	0.554	.	.	.	.		848	1238
128	Mumcu G	Utilisation of dental services in Turkey: a cross-sectional survey	2004	Turkey	UMI	Cross-sectional	Random Sampling	Quantitative logistic regression	Urban and rural communi	NR	Adult	Patients	866	350	0.404	Curative base	Private dental	516	0.596	Mostly 20-29	371	495

									ty based survey							d dental care	services					
129	Nagarjuna P	Utilization of dental health-care services and its barriers among the patients visiting community health centers in Nellore District, Andhra Pradesh: A cross-sectional, questionnaire study	2016	India	LMI	Cross-sectional	Multistage cluster sampling	Dectriptive Quantitative	Community Health Centres	NR	General adult dental patient	Patients	600	216	0.36	Curative based dental care	Government based CHCs dental services	384	0.64	Mean 35 ± 7.5	347	253
130	Nagdev P	Andersen health care utilization model: A survey on factors affecting the utilization of dental health services among school children.	2023	India	LMI	Cross-sectional	Consecutive sampling	Multiple logistic regression analysis	School-based survey	Andersen's Model	Adolescents	Parent	1100		0.219	.	Government hospital (24.7%), Private clinics (13.9%)	572	0.52	13-15	582	518
131	Nakyonyi MG	Use of dental care services among adolescents living with HIV on antiretroviral treatment in Kampala, Uganda: a cross-sectional study.	2024	Uganda	LI	Cross-sectional	Convenience sampling	modified Poisson regression for multivariate analysis	Health service centre	Andersen's Model	Adolescents with HIV on ART	Self-reported	154		0.123	Tooth extraction	.	.	0.877	10-18	89	65
132	Nasir EF	Utilization of dental health care services in context of the HIV epidemic- a cross-sectional study of dental patients in the Sudan.	2009	Sudan	LI	Cross-sectional	Consecutive sampling	Logistic regression	Health service centre	Andersen's Model	General adults	Self-reported	1262	.	0.539	.	.	.	.	30.7 ± 8.5	548	712
133	Nija MAG	Oral health care-seeking behaviour and influencing factors among 18-34 years old women in Kochi, India	2020	India	LMI	Cross-sectional	Convenience Sampling	Quantitative Descriptive and Chi-Square Test	Tertiary dental care hospital	NR	Women	Patients	194	132	0.68	Curative based dental care	Private based dental services	2	0.01	Mean 27.1 ± 5.2	NA	194
134	Obeidat SR	Factors influencing dental care access in Jordanian adults.	2014	Jordan	UMI	Cross-sectional	Convenience sampling	Descriptive statistics	Public health centre	NR	General adults	Self-reported	614	573	0.933	Emergency (89%)	Private (79.6%), Public (20.4%)	41	0.067	18-65	282	332

135	Ocwia J	Oral health seeking behaviors of adults in Nebbi District, Uganda: a community-based survey.	2021	Uganda	LI	Cross-sectional	Simple random sampling	Multivariable logistic regression	Community based survey	NR	General adults	Self-reported	400	106	0.52	Toothache (86.7%)	.	.	.	24-43 (median 32)	170	230
136	Ogbuji OS	Pattern of utilization of dental services among Nigerian population in the University of Benin community: A prospective study	2023	Nigeria	LMI	Cross-sectional	Convenience Sampling	Quantitative Descriptive	University based survey	NR	University Staff	Patients	197	25	0.127	Curative based dental care	Private based dental services	82	0.416	25-55	102	95
137	Ojok S	Factors associated with utilization of oral health services among adults aged 18-70 years in Lira district, Northern Uganda: a community based cross-sectional study.	2024	Uganda	LI	Cross-sectional	Multistage cluster sampling	Univariate, bivariate, and multivariate analysis	Community based survey	NR	General adults	Self-reported	576	118	0.205	.	.	.	.	18-70	283	293
138	Okoroafor CC	Dental Health Knowledge Attitude and Practice Among University of Calabar Students	2023	Nigeria	LMI	Cross-sectional	Multistage randoms ampling	Quantitative with structured questionnaire	University based survey	NR	Students	Patients	430	118	0.274	Curative based dental care	Private based dental services	312	0.726	20-23	191	239
139	Oliveira ACB	Parental acceptance of restraint methods used for children with intellectual disabilities during dental care	2007	Brazil	UMI	Cross-sectional	Convenience Sampling	Quantitative Descriptive and Chi-Square Test	Community institutions for children with intellectual disabilities	NR	Children with intellectual disabilities	Caregiver/Parents	209	171	0.82	Dental care requiring behavioral management techniques	Private based dental services	38	0.18	5-26	138	71
140	Oliveira ACB	Mothers' perceptions concerning oral health of children and adolescents with Downsyndrome: A qualitative approach	2010	Brazil	UMI	Qualitative exploratory study	Purposive sampling	Thematic content analysis of in-depth interviews	Public hospital	Grounded in socio-cultural and biomedical	Children/adolescents with Down syndrome	Mother	19	NR	NR	Curative based dental care	NR	NR	NR	3-15	NA	NR

										interpretations of health												
141	Onyejaka NK	Barriers and facilitators of dental service utilization by children aged 8 to 11Â years in Enugu State, Nigeria.	2016	Nigeria	LMI	Cross-sectional	Multistage stratified sampling	Bivariate analysis	School-based survey	NR	Children	Parent	1406	116	0.147	.	.	.	.	8-11 (9.32 ± 1.08)	672	734
142	Oredugba FA	Use of oral health care services and oral findings in children with special needs in Lagos, Nigeria.	2006	Nigeria	LMI	Case-control	NR	Percentages	School-based survey	NR	Children with Special Needs	Parent	243	.	.	.	.	.	.	12.6+3.4	128	115
143	Osuh ME	Oral health in an urban slum, Nigeria: residents' perceptions, practices and care-seeking experiences.	2023	Nigeria	LMI	Qualitative	NR	Thematic analysis	Focus group discussion	NR	General adults	Self-reported	58	.	.	.	.	.	.	25-59	29	29
144	Ou XY	[Status and strategies of oral health service demand and medical treatment utilization among 3- to 5-year-old preschool children in Jiangxi province].	2018	China	UMI	Cross-sectional	Stratified cluster sampling	Logistic regression	Community based survey	NR	Children	Parent	2880	266	0.1731	Checkup (57.63%), Preventive (28.81%)	.	1271	0.8269	3-5	1488	1392
145	Patel KH	Cultural and Socioeconomic Barriers in Utilization of Dental Services: A Cross Sectional Questionnaire Based Study	2016	India	LMI	Cross-sectional	Convenience sampling	Quantitative with structured questionnaire	Outpatient department of a private dental institution includes satellite dental center, and private clinics	NR	General adults	Self-reported	404	NR	NR	Curative based dental care	Private basedv Dental services	232	NR	NR	232	172
146	Pengpid S	Dental service utilization in the general adult population in Bangladesh.	2024	Bangladesh	LMI	Cross-sectional	Multistage probabilit	Hierarchical multivariable analysis	Population based survey	NR	General adults	Self-reported	8185		0.159	Curative	Private clinics (35.1%), Medicine shop (25.1%),		0.71	18–69 (36.6 ±14.3)	3804	4381

							y-based sampling										Village doctor (19.7%)					
147	Phoosuwan N	Oral health knowledge, literacy and behavior of pregnant women: a qualitative study in a northeastern province of Thailand.	2024	Thailand	UMI	Qualitative	Purposive sampling	Percentages	Health service centres	NR	Pregnant women	Self-reported	20	17	0.85	Gum problem				18–43(26)		20
148	Pinto RS	[Characteristics associated with the use of dental services by the adult Brazilian population].	2012	Brazil	UMI	Cross-sectional	Probability sampling	Multivariate analysis	Population based survey (SB Brasil 2003)	Andersen and Newman model	General adults	Self-reported	12425			Pain or maintenance	Public services (51.8%), Private services (37.1%)	378	.	35-44 (39.2 ± 3.2)	3947	8478
149	Piovesan C	Individual and contextual factors influencing dental health care utilization by preschool children: a multilevel analysis	2017	Brazil	UMI	Cross-sectional	Random sampling	Quantitative with structured questionnaire	Public health centers	NR	Preschool children	Care giver/Parents	639	138	0.216	Curative based dental care	Public Health Centres	497	0.784	1–5	321	318
150	Pontigo-Loyola AP	[Influence of predisposing, enabling, and health care need variables on the use of dental health services among Mexican adolescents from a semi-rural location].	2012	Mexico	UMI	Cross-sectional	Stratified sampling	Multivariate analysis	School-based survey	Andersen's Model	Adolescents	Self-reported	1538	231	0.15	.	.	.	.	12-15	770	768
151	Poudyal S	Utilization of dental services in a field practice area in Mangalore, Karnataka	2010	India	LMI	Cross-sectional	Random sampling	Questionnaire based survey	Household survey	NR	General adults	Self-reported	182	122	0.67	Tootache		52	0.286	18-70 (36.5 ± 13.4)	62	120
152	Pradeep Y	Gaps in need, demand, and effective demand for dental care utilization among residents of Krishna district, Andhra Pradesh, India.	2016	India	LMI	Cross-sectional	Cluster random sampling	Percentages	Rural and Urban Community survey	NR	General adults	Self-reported	3102	676	0.214			2426	0.786	15 and above	1303	1799
153	Prasanth P	Utilization of Dental Health Care Services among 12 Year School Going Children of Nellore City, Andhra Pradesh, India-Across Sectional Study	2019	India	LMI	Cross-sectional	Random sampling	Quantitative with structured questionnaire	School based Survey	NR	School going children	Care giver/Parents	323	80	0.248	Curative based dental care	NR	243	0.752	12	151	172

154	Pruksanusak W	Association of religious and socio-cultural factors on dental service utilization among the elderly in Narathiwat, Thailand.	2024	Thailand	UMI	Cross-sectional	Purposive sampling	Quantitative (face-to-face interviews, descriptive and chi-square analysis)	Public dental services	Penchansky & Thomas's Five A's of Access	Older adults	Self-reported	106	NR	NR	Curative based dental care	Public dental services	NR	NR	60–88	47	59
155	Puthiyapurayil J	Parental perception of oral health related quality of life and barriers to access dental care among children with intellectual needs in Kottayam, central Kerala-A cross sectional study.	2022	India	LMI	Cross-sectional	Simple random sampling	Descriptive analysis	Health service centres	NR	Children with intellectual disabilities	Parent	300			Emergency visits				4-12 (8.05 ± 1.87)	144	156
156	Qi X	Urban-Rural Disparities in Dental Services Utilization Among Adults in China's Megacities.	2021	China	UMI	Cross-sectional	Multistage stratified sampling	Multivariate logistic regressions	Megacities based survey	Andersen's Model	General adults	Self-reported	4049	2817	0.695			1232	0.3042	18–65	.	.
157	Rahebi D	Utilization of dental care in Iranian pregnant women: Findings from a population-based study.	2021	Iran	UMI	Cross-sectional	Random sampling	Percentages	Population based survey	NR	Pregnant women	Self-reported	4071	1846	0.4535			2225	0.5465	15 and above	.	4071
158	Rajput S	Oral health perceptions, behaviors, and barriers among differently abled and healthy children.	2021	India	LMI	Cross-sectional	Cluster random sampling	Descriptive analysis	School-based survey	NR	Differently abled children	Parent		78	0.26	Pain		222	0.74	7.0-15	.	.
159	Rambabu T	Reasons for use and nonuse of dental services among people visiting a dental hospital in urban India: A descriptive study.	2018	India	LMI	Cross-sectional	Deliberate sampling	Percentages	Hospital-based survey	NR	General adults	Self-reported	1800	1155	0.6417	Dental pain		645	0.3583	18-65	860	940
160	Rana BK	Assessment of treatment needs, barriers, and self-perception regarding oral health among female university students: a cross-sectional study.	2024	Pakistan	LMI	Cross-sectional	Convenience sampling	Descriptive analysis	University survey	NR	University going females	Self-reported	400	95	0.237					18-22 (19.91 ±1.22)	.	400
161	Rebelo Vieira JM	Contextual and individual determinants of non-utilization of dental services among Brazilian adults.	2019	Brazil	UMI	Cross-sectional	Multistage sampling	Multilevel logistic regression analysis	Population based survey (Brazilian Oral Health Survey 2010)	Andersen's Behavioral Model	General adults	Self-reported	7265	.	.	.	.	.	0.047	35-44	2396	4869
162	Reddy LS	Self-Reported Obstacles to Regular Dental Care among	2016	India	LMI	Cross-sectional	NR	Multiple Logistic	IT company survey	NR	General adults	Self-reported	1017	429	0.47					20 and above	574	443

		Information Technology Professionals.						Regression analysis														
163	Rezaei S	Dental health-care service utilisation and its determinants in West Iran: a cross-sectional study	2018	Iran	UMI	Cross-sectional	Multistage sampling	Quantitative self-administered questionnaire	Urban community based survey	Andersen's Behavioral Model	General Adults	Patients	894	540	0.603	Emergency and Need Based Dental Care	Public dental services	354	0.397	Mean 44.6 ± 12.1	835	59
164	Rezaei S	Socioeconomic-related inequalities in dental care utilization in northwestern Iran	2020	Iran	UMI	Cross-sectional	Cluster random sampling	Quantitative – structured face-to-face interviews using validated questionnaire	Community-based, household survey	Andersen's Behavioral Model	General adults	Patients	436	258	0.592	Emergency and Need Based Dental Care	Public dental services	178	0.408	Mean 46.4	394	42
165	Rezaei S	Socioeconomic inequality in dental care utilization in Iran: A decomposition approach	2019	Iran	UMI	Cross-sectional	3-stage stratified cluster sampling	Econometric decomposition using concentration index and regression analysis	Urban and rural households	Concentration index and Wagstaff decomposition model	General adults	Patients	37860	1767	0.0467	Curative based dental care	Public dental services	36094	0.9533	Mean 51.7 ± 15.5	32595	5265
166	Rezaei S	Dental care utilization in the west of Iran: a cross-sectional analysis of socioeconomic determinants	2016	Iran	UMI	Cross-sectional	Multistage sampling	Multivariable logistic regression	Household survey	NR	General adults	Self-reported	520	319	0.613	Restorations				23-79 (41.9 ± 12.3)	483	37
167	Riaz A	Knowledge, attitude, and practices of pregnant women regarding oral health at railway hospital rawalpindi, pakistan	2020	Pakistan	LMI	Cross-sectional	Convenience sampling	Frequencies and percentages	Health service centres	NR	Pregnant women	Self-reported	260	140	0.538	Bleeding gums		120	0.462	18-30		
168	Rocha-Buelvas A	Barriers of access to oral health care among university students in southern Colombia, 2011. A multivariate analysis	2014	Colombia	UMI	Cross-sectional	Stratified probability sampling	Quantitative self-administered questionnaire	University-based	Andersen's Behavioral Model	Undergraduate university students	Patients	338	193	.	Curative based dent	Public and private mixed	145	0.429	20–24	143	195

																al care						
169	Rocha-Buelvas A	Oral health services use among schoolchildren/teens with developmental disabilities in Colombiaâ€™s capital district, 2015	2019	Colombia	UMI	Cross-sectional	Simple random sampling	Quantitative self-administered questionnaire	Special education schools	Aday & Andersen’s Behavioral Model	School children/adolescents with intellectual disabilities	Caregiver/Parents	102	73	0.716	Curative based dental care	NR	29	0.289	4–18	68	34
170	Rodrigues A	An exploration of the oral health beliefs and behaviors of people living with HIV in Mangalore, India: a qualitative study.	2021	India	LMI	Qualitative	Convenience sampling	Content analysis	Health service centres	NR	HIV-positive adults	Self-reported	16			Curative				44.93 ± 8.8	9	7
171	Rodrigues, LAM	The use of dental services among preschool children: a population-based study	2014	Brazil	UMI	Cross-sectional	Cluster sampling	Logistic regression	Community based survey	Andersen’s behavioral model	Pre-school Children	Parent/Caregiver	809	126	0.155	Complaints (Gingival bleeding, Pain, Caries)	Public services (59.5%), Non-public services (40.5%)	682	0.845	18-36 months	401	408
172	Sachedina T	Reasons for Delay in Seeking Treatment for Dental Caries in Tanzania.	2023	Tanzania	LMI	Cross-sectional	Stratified random sampling	Frequencies and percentages	Health service centres	NR	General adults	Self-reported	315							18-81 (37)	134	181
173	Saddki N	Factors associated with dental visit and barriers to utilisation of oral health care services in a sample of antenatal mothers in Hospital Universiti Sains Malaysia.	2010	Malaysia	UMI	Qualitative	Systematic random sampling	Logistic regression analysis	Health service centres	NR	Pregnant women	Self-reported	124	36	0.29	Curative	Private service (58.3)			19-45 (31.1± 5.81)		
174	Saengtipbovorn S	FACTORS ASSOCIATED WITH THE UTILIZATION OF DENTAL HEALTH SERVICES BY THE ELDERLY PATIENTS IN HEALTH CENTER NO.54, BANGKOK, THAILAND	2012	Thailand	UMI	Cross-sectional	Systematic sampling	Quantitative face-to-face interviews using a validated structured questionnaire	Urban health center	PRECEDE framework	Elderly	Patients	335	164	0.489	Emergency and Need Based Dental Care	Health Center	171	0.51	Mean 66.5 ± 5.9	80	255

175	Saleh NMH	Barriers Affecting the Utilization of Dental Health Services among Community Dwelling Older Adults	2018	Egypt	LMI	Cross-sectional	Multistage random sampling	Quantitative with structured questionnaire	Geriatric outpatient departments, urban public hospitals	Andersen Behavioral Model	Older adults	Patients	612	270	0.441	Emergency and Need Based Dental Care	Public dental clinics	342	0.559	60–89	331	281
176	Samuel SR	Transgender HIV status, self-perceived dental care barriers, and residents' stigma, willingness to treat them in a community dental outreach program: Cross-sectional study.	2018	India	LMI	Cross-sectional	Chain referral sampling	Descriptive statistics	Community based survey	NR	Transgender adults	Self-reported	190	9	0.047			181	0.953	28.6 ± 5.5		
177	Schroeder FMM	Oral health condition and the use of dental services among the older adults living in the rural area in the south of Brazil.	2020	Brazil	UMI	Cross-sectional	Systematic random sampling	Descriptive analysis	Rural population based survey	NR	Older adults	Self-reported	1030		0.139	Emergency visits	Private service (69.74), Public health service (13.33), Convenant (8.04), Health plan (10.31)		0.066	60 and above	568	462
178	Sekele PM	Frequency and factors associated with the utilization (curative and preventive) of oral health care services among pregnant women in Kinshasa, Democratic Republic of Congo.	2025	Congo Dem. Rep.	LI	Cross-sectional	Simple random sampling	Multivariate regression analysis	Hospital-based survey	NR	Pregnant women	Self-reported	500	25	0.05	Dental pain	.	.	.	28.4±5.3		500
179	Sermsuti-Anuwat N	Perspectives and experiences of Thai adults using wheelchairs regarding barriers of access to dental services: a mixed methods study.	2018	Thailand	UMI	Mixed Methods	Convenience sampling	Multivariate analysis, Thematic analysis	Health service centre	Penchansky and Thomas	Adults with wheelchairs	Self-reported	156	.	.	.	.	.	.	21-62 (median: 49)	113	43
180	Sevik I	Understanding poor oral health among older adults in Turkiye: socioeconomic and healthcare access challenges.	2025	Turkey	UMI	Cross-sectional	Stratified, two-stage cluster sampling	Percentages	Community based survey	NR	Older adults	Self-reported	3144	2364	0.752	.	.	.	.	mean 72.57	1445	1699
181	Sharma A	Oral hygiene practices and factors affecting oral health service utilization among children (11-14 years) of government	2019	India	LMI	Cross-sectional	Convenience sampling	Quantitative with structured questionnaire	Government school, urban	NR	Children	Patients	200	61	0.305	Curative based	Public dental services	139	0.695	11–14	92	108

		school of nikol ward of East Zone of Ahmedabad, Gujarat, India														dental care						
182	Sidharthan S	Utilization of dental health services and its associated factors among adult population in Ernakulam district, Kerala, India: A mixed-method analysis.	2024	India	LMI	Mixed Methods	Cluster sampling	Thematic analysis	Community based survey	Andersen Health care Model for Health care Utilization	General adults	Self-reported	544	379	0.6966	Having trouble with teeth	.	165	0.3033	18 to 78 (49.8 ± 15.9)	232	312
183	Silva ELM SD	Oral health care for children and adolescents with cerebral palsy: perceptions of parents and caregivers.	2020	Brazil	UMI	Cross-sectional	Convenience sampling	Descriptive analysis	Health service centres	NR	Children and adults with cerebral palsy	Parent/ care giver	94	86	0.915	Checkup	Public service (64.7), Private service (35.3)	8	0.085	5.0-18.0	61	33
184	Songa MAS	Prevalence of Dental Caries and Periodontal Disease, Access to Dental Services and Perception of Oral Health in Adolescents and Adults from a Rural Community in Angola.	2024	Angola	LMI	Cross-sectional	Convenience sampling	Descriptive statistics	Rural community based survey	NR	General adults	Self-reported	575	173	0.3009	Preventive consultation	Public service (85.11), Private service (8.21), Faculty (2.74), Others (3.95)	246	0.428	22.36 ± 9.07	323	252
185	Soria GS	[Access to and use of oral health services among the elderly in Pelotas, Rio Grande do Sul State, Brazil].	2019	Brazil	UMI	Cross-sectional	Two-stage cluster sampling	Poison regression	Community based survey	Andersen's Behavioral Model	Older adults	Self-reported/ Care giver	1451	524	0.383	Routine (35.6%), pain (9.9%), lesions	private clinics (84.2%)	45	0.031	>60	537	914
186	Subedi K	Oral health status and barriers to utilization of dental services among pregnant women in Sunsari, Nepal: A cross-sectional study.	2024	Nepal	LMI	Cross-sectional	Purposive sampling	Percentages	Health service centre	NR	Pregnant women	Self-reported	139	7	0.05	.	.	132	0.95	18-35 (23.60 ± 2.99)	.	139

187	Sujlana A	Barriers of dental care utilization for children living in military and civilian areas.	2016	India	LMI	Cross-sectional	Systematic random sampling	Multivariate regression analysis	School-based survey	NR	School children	Parent via survey and children via clinical examination	400	.	.	.	.	.	.	4.5-5.5		
188	Sun W	The routine utilization of dental care during pregnancy in eastern China and the key underlying factors: A Hangzhou city study	2014	China	UMI	Cross-sectional	Convenience sampling	Quantitative with structured questionnaire	Urban health institutions	Andersen's Behavioral Model	Pregnant women	Patients	2259	367	0.1673	Emergency and Need Based Dental Care	Public dental services	1892	0.832	Mean 29.3 ± 3.4	NA	2259
189	Suprabha BS	Early Childhood Caries and Dental Care Utilization in Mangalore, India: Parents' Perceptions.	2024	India	LMI	Qualitative study	Purposive sampling	Qualitative appraoch	Health service centres	NR	Children with ECC	Parent	27	.	.	Swelling and pain	.	.	.	35.15 ± 6.12		
190	Supriya	Relevance of Emotion of Anxiety and Fear of Dentistry as Motivational Conflict in Oral Health Behaviors.	2024	India	LMI	Cross-sectional	Convenience sampling	Quantitative approach	Health service centres	NR	General adults	Self-reported	84	.	.	.	.	.	.	20-40 (29.083 ± 6.381)	39	45
191	Suresh LR	Barriers to utilisation of dental services among children with disabilities in a coordinated healthcare programme in mangalore in india: A mixed methods study	2020	India	LMI	Mixed Methods	Purposive sampling	Interpretative phenomenological approach	Health service centres	NR	Children with special healthcare needs	Parent	114	32	28.07	.	.	.	.	5-21 (12.5 ± 3.21)	69	45
192	Susarla SM	Cross-Sectional Analysis of Oral Healthcare vs. General Healthcare Utilization in Five Low- and Middle-Income Countries	2022	Ecuador, El Salvador, India ,	LMI	Cross-sectional	Convenience sampling from a community-based oral	Quantitative Structured interviews with mothers	Mixed (urban, rural, peri-urban) across 5 countries	Andersen's Behavioral Model	Mothers and their children	Patients	7746	3917	76.4% mothers, 30.1%	Emergency and Need Based	Public dental services	3829	23.6% mothers, 30.	Mothers 25–35 , Children 6 month	NA	3422 mothers

				Nepal, Vietnam			health program								children	Dental Care			1% children	s- 6 years		
193	Tan YR	Demographic and socioeconomic inequalities in oral healthcare utilisation in Malaysia: evidence from a national survey.	2021	Malaysia	UMI	Cross-sectional	Cluster sampling	Logistic regression analysis	National household survey	Andersen's Behavioural Model.	General adults	Self-reported	11308		13.2	Preventive in young and curative in elderly				18-34	5345	5963
194	Tefera AT	Dental health problems and treatment-seeking behavior among special need school students in Amhara region, Ethiopia.	2021	Ethiopia	LI	Cross-sectional	Simple random sampling	Bivariable and multivariable logistic regression	School-based survey	NR	Children with special healthcare needs	Self-reported	443	123	60.29	Serious stage of dental problem				7-30 (15.84 ±0.18)	237	206
195	Telang LA	Exploring the barriers and enablers of oral health care utilisation and safe oral sex practices among transgender women in Malaysia: a qualitative study.	2025	Malaysia	UMI	Qualitative	Snow-ball sampling	Thematic analysis	Community based survey	NR	Transgender adults	Self-reported	20	4	20	Aesthetics		16	80	39.8		
196	Tharshini P	Assessment of Various Factors Contributing to Unfavourable Oral Health Care Seeking Behaviour: A Cross-Sectional Study.	2024	India	LMI	Cross-sectional	Multistage random sampling	Multinomial logistic regression analysis	Community survey	Thorpe et al/ Penchansky and Thomas	General adults	Self-reported	624	.	.	.	.	.	0.1	30-55	312	312
197	Turton M	Oral health care experiences of people living with HIV in Kwazulu-Natal and Western Cape, South Africa	2015	South Africa	UMI	Cross-sectional	Non-probability sampling	Binary logistic regression	Health service centres	NR	HIV-positive adults	Self-reported	435	288	66.2	Curative				18 and above		
198	Uguru N	Oral health-seeking behavior among different population groups in Enugu Nigeria.	2021	Nigeria	LMI	Cross-sectional	Multistage sampling	Frequency and percentages	Population based survey	Anderson and Newman	General adults	Self-reported	774			Pain				21-40	326	448
199	Vadher R	Assessment of Caries-Prevalence, Unmet Dental Treatment Needs and Barriers to Care as Perceived by Parents of Private and Government Aided-School Children of Gandhinagar-An Epidemiological Study	2024	India	LMI	Cross-sectional	Sratified random sampling	Percentages	School-based survey	NR	Children	Parent	750	.	.	.	.	.	.	6-12		

200	Vali L	Investigating the access barriers to oral and dental health services for children from the perspective of parents attending the health centers of Kerman.	2023	Iran	UMI	Cross-sectional	Cluster sampling	Percentages	Health service centre	NR	Children	Parent	410	.	.	.	.	.	.	5-16 (8.83± 2.88)	195	215
201	Varenne B	Reasons for attending dental-care services in Ouagadougou, Burkina Faso	2005	Burkina Faso	LI	Cross-sectional	Random sampling	Quantitative with structured questionnaire	Urban – public, private, and NGO dental facilities	Andersen Model	General dental patients	Patients	14591	14591	NR	Emergency and Need Based Dental Care	Public/ Private and NGO dental services	7895	0.557	15–44	6822	7343
202	Vargas NV	Association between access to dental services and altitude of the residence of Peruvian children	2023	Peru	UMI	Cross-sectional	NR	Poisson regression	Nationwide survey (secondary data)	Andersen's Behavioral Model	Children	Parent	42115	19092	0.4533		Public services	19823	0.4707	0-11	.	.
203	Varsha MR	A Cross-Sectional Study to Assess the Perceived Oral Health Care Needs, Barriers to Accessing Oral Health Care Services, and Utility Among the Rural Population in Chengalpattu District, Tamil Nadu.	2024	India	LMI	Cross-sectional	Stratified random sampling	Percentages	Rural Household survey	NR	General adults	Self-reported	570	241	37.5	Toothache		37	33.3	18 and above	248	322
204	VÃsquez-HernÃndez	Perception About Oral Health and Its Determinants in Women in Context of Prostitution in MedellÃn (Colombia): Expressions of Social Vulnerability	2018	Colombia	UMI	Qualitative	Purposive sampling	Thematic content analysis	Community- based survey	NR	Women (Sex-workers)	Self-reported	12	NR	NR	Pain, Broken teeth	NR	NR	NR	20-80	.	12
205	Verma H	Access to public dental care facilities in Chandigarh.	2012	India	LMI	Cross-sectional	Multistage random sampling	Percentages	Community survey	NR	General adults	Self-reported	203	.	.	.	.	.	.	43 ± 14.4	99	104
206	Villalobos-Rodelo	Dental Needs and Socioeconomic Status Associated with Utilization of Dental Services in the Presence of Dental Pain: A Case-Control Study in Children	2009	Mexico	UMI	Case-control study	Random sampling	Quantitative with structured questionnaire	School-based survey	NR	Children attending school	Caregiver/Parents	1516	NR	NR	Routine Dental Check up	School based dental care	NR	NR	Mean 9.01 and 8.65	687	829
207	Vuyyuru CR	Dental Diseases and Factors Defining Utilization of Dental Care Services among Rural	2021	India	LMI	Cross-sectional	Multistage	Descriptive statistics using	Household survey	NR	Children of rural area	Self-reported	169		12	Curative		88	52.07	12	88	81

		Children Aged 12 Years in Nellore District, Andhra Pradesh: A Community-Based Study.					Random sampling	Frequency tables														
208	Wanichsaithong P	Predictors of dental service utilization among visually impaired people in Chiang Mai, Thailand	2015	Thailand	UMI	Cross-sectional	Convenience sampling	Quantitative with structured questionnaire	Community-based Survey	Andersen's Behavioral Model of Health Service Utilization	Adults and elderly with visual impairment	Patients	266	70	0.263	Emergency and Need Based Dental Care	Private based dental services	196	0.737	35–44 & 60–74	NR	NR
209	Wei LJ	Oral health self-management barriers among rural older adults in Guangxi, China: A qualitative study.	2024	China	UMI	Qualtitative	NR	Colaizzi's phenomenological method	Rural Household survey	NR	Older adults	Self-reported	25	NR	NR	NR	NR	NR	NR	60-80	.	.
210	Widita I	Factors Associated with Dental Care Utilization among Pregnant Women at Klapanunggal Health Centre, Bogor, Indonesia	2019	Indonesia	UMI	Cross-sectional	Convenience sampling	Quantitative with structured questionnaire	Health Service Center	NR	Pregnant women	Patients	60	14	0.233	Emergency and Need Based Dental Care	Public dental services	46	0.767	<35 years	.	60
211	Wu B	Dental service utilization among urban and rural older adults in China - A brief communication	2007	China	UMI	Cross-sectional	Stratified random sampling	Logistic regression	Rural and urban communities	Andersen's behavioral model	Older adults	Self-reported	1044	70	0.067	NR	NR	NR	NR	60-103	449	595
212	Yaghoubi Z	Inequalities in Unmet Oral Health Care Need Among Adults in East Iran, a Cross Sectional Population-Based Study	2021	Iran	UMI	Cross-sectional	Stratified random sampling	Quantitative with structured questionnaire	Urban and suburban Community-based survey	Bradshaw's model of health needs	General adult	Patients	1475	NR	NR	Emergency and Need Based Dental Care	Public dental services	NR	NR	Mean 39	445	1029
213	Zardak AN	Factors associated with dental care utilization among Iranian	2025	Iran	UMI	Cross-sectional	Multistage sampling	Simple logisitc regression	Household survey	Anderson's behavi	General adults	Self-reported	704	420	59.7	Curative	NR	NR	NR	18-44	302	402

		adult populations based on Anderson model.								oural model						(97.1)						
21 4	Zenci rciog lu, Ã–K	Access to health care after dental trauma in children: A quantitative and qualitative evaluation	20 19	Turk ey	UMI	Mixed Metho ds	Maximu m variation sampling	Logistic regression analysis	Health service centre	NR	Children with dental trauma	Pare nt	14 0	NR	NR	NR	NR	NR	NR	7.6 ± 2.7	98	42

UMI: Upper-middle income, LMI: Lower-middle income, LI: Low-income, NR: Not reported
